# New insights for *Drosophila* GAGA factor in larvae

**DOI:** 10.1098/rsos.150011

**Published:** 2015-03-18

**Authors:** Marta Blanch, David Piñeyro, Jordi Bernués

**Affiliations:** 1 Departament de Genòmica Molecular, Institut de Biologia Molecular de Barcelona-CSIC, Parc Científic de Barcelona, Barcelona 08028, Spain; 2 Cell and Developmental Biology Programme, Institute for Research in Biomedicine, Barcelona, Spain

**Keywords:** GAGA factor, gene regulation, transcriptome analysis

## Abstract

GAGA factor plays important roles during *Drosophila* embryogenesis and its maternal contribution is essential for early development. Here, the role of GAGA factor was studied in 3rd instar larvae using depletion and overexpression conditions in wing disc and transcriptome analysis. We found that genes changing expression were different to those previously described using GAGA mutants in embryos. No apparent phenotypes on GAGA depletion could usually be observed at larval stages in imaginal discs but a strong effect on salivary gland polytene chromosomes was observed. In the adult, GAGA depletion produced many defects like abnormal cell proliferation in the wing, impaired dorsal closure and resulted in homeotic transformation of abdominal segment A5. Unexpectedly, no effects on Ultrabithorax expression were observed. Short overexpression of GAGA factor in 3rd instar larvae also resulted in activation of a set of genes not previously described to be under GAGA regulation, and in lethality at pupa. Our results suggest a little contribution of GAGA factor on gene transcription in wing discs and a change of the genes regulated in comparison with embryo. GAGA factor activity thus correlates with the global changes in gene expression that take place at the embryo-to-larva and, later, at the larva-to-pupa transitions.

## 2. Introduction

GAGA factor is the product of the Trithorax-like gene, *Trl*, in *Drosophila* [1]. By binding to its DNA recognition sequence (consensus GAGAG) [2], this transcription factor (TF) has been described to modulate the expression of several genes involved in different processes. Among them are homeotic genes and developmental genes: Ultrabithorax (*Ubx*), engrailed (*en*), fushi-tarazu (*ftz*), even-skipped (*eve)*, etc. [3–8]. However, several studies indicated a more complex picture by showing GAGA involved in chromatin remodelling, in Polycomb responsive element (PRE) function, and in insulator/boundary functions [5,9–14]. On the other hand, the presence of GAGA factor on the promoter regions of a relatively high number of genes may explain its highly pleiotropic effects [15,16]. The fact that *Trl* is an essential gene that presents a strong maternal effect added difficulty to its study [17]. Thus, most of the GAGA target genes have been inferred from the phenotypes obtained by the use of hypomorph and/or hypomorph/null *Trl* allele combinations but almost no evidence for direct effects have been shown for these genes. *Trl* is expressed as a constitutive gene in all stages and, probably, all tissues of the fly, albeit mRNA levels change notably [18]. Most of the studies performed to date concentrate on the roles of GAGA factor at embryo stages but very little is known about its role at later stages (larval, adult). Here we have addressed the functions of *Trl* at 3rd instar larval stage by using a combination of knockdown and overexpression experiments. A set of genes whose expression appears modulated by GAGA factor levels has been identified in 3rd instar larvae that were not previously described. Together with phenotypic analysis these results suggest a different contribution for GAGA factor in larval wing discs and salivary glands.

## 3. Material and methods

### 3.1 Fly stocks

Homozygous stocks *Tub*GAL80^ts^; UAS GAGA, *Tub*GAL80^ts^; UAS GFP, UAS DIAP1; UAS GAGA and *Nubb*GAL4; UAS*Dicer2*, were obtained by conventional crosses with balanced stocks. *Act*GAL4/TM6b, *dpp*GAL4/TM6b, *ptc*GAL4, 69BGAL4 were obtained from Bloomington Stock Center. Transgenic homozygous UAS GAGA_RNAi_ (II) was obtained by microinjection as described [19]. GAGA RNAi fly stocks numbers 41095 and 17198 were obtained from Vienna Drosophila RNAi Center (VDRC, Vienna). Flies were propagated at 25°C as usual and shifted to the indicated temperatures whenever required. While GAGA factor is expressed in several isoforms [20]; in this work, all transgenic constructs overexpressed only the GAGA_519_ isoform and all GAGA_RNAi_ constructs knocked down the expression of all GAGA isoforms (as in Bernués *et al.* [19]).

### 3.2 Total RNA preparation and microarray analysis

For transcriptome analysis of GAGA overexpression, *Ac*tGAL4/TM6b females were crossed with *Tub*GAL80^ts^; UAS GAGA males at 18°C until larvae began to wander out of the food. At this point, they were shifted to 29°C for approximately 13 h to allow GAGA overexpression. Non-tubby larvae (i.e. those carrying the ActGAL4) were selected at this time and wing discs were transferred to eppendorf tubes. The same was performed in parallel for a control cross in which a *Tub*GAL80^ts^; UAS GFP was used. For transcriptome analysis of GAGA knockdown 69BGAL4 homozygous females were crossed with homozygous UAS GAGA_RNAi_ males. For these experiments, a strain of GAGA RNAi different to the one previously reported was used because a homozygous strain allowed unambiguous identification of larvae after crossing with 69BGAL4 flies (the previous one was heterozygous). In all cases, even using several additional GAGA RNAi strains (VDRC stock numbers 41095 and 17198), all the phenotypes observed here and before were identical ([19] and results not shown). In parallel, a control cross using homozygous UAS GFP_RNAi_ males was performed. At 3rd instar stage, larvae were dissected, 25–30 wing discs collected on Schneider's cell culture medium and total RNA was extracted using Trizol (500 μl, Ambion). Discs were thoroughly homogenized using a small plastic pestle. After 5 min at room temperature, 100 μl of chloroform were added, and after 10 s vortexing the mix was allowed to settle for 5 min at room temperature. After 15 min spinning at 13 000 r.p.m. at 4°C, the upper phase (170 μl) was recovered and further purified using the RNAeasy mini kit (Qiagen) following the manufacturer's indications. Total RNA was ethanol precipitated and resuspended in RNAse free water. RNA amount and quality were assessed using Nanodrop and Bioanalyzer apparatus and further processed for transcriptome analysis at the IRB Genomics facility (Barcelona). The results were normalized and analysed at the Biostatistics & Bioinformatics Unit (IRB Barcelona) using a robust multi-array average algorithm to prepare data for analysis [21]. For pairwise comparisons, moderated *t*-test statistics [22], as implemented in the Bioconductor library limma, were used. The probability that each gene was differentially expressed was computed by fitting a semi-parametric partial *t* density [23], and a list of differentially expressed genes was obtained by controlling the Bayesian false discovery rate (FDR) below 5% [24]. Differential expression was obtained after subtraction of the respective GFP controls.

Gene ontology analysis was carried out using the DAVID package from NIH at high stringency conditions (Database for Annotation, Visualization, and Integrated Discovery, http://david.abcc.ncifcrf.gov/home.jsp). Among the different types of analysis in the DAVID program, we have used the Functional Annotation Clustering and the Functional Annotation Chart. Results are presented as a function of the Enrichment Score that is the geometric mean (in –log scale) of member's *p*-values (EASE score) in a corresponding annotation cluster and is used to rank their biological significance. It is an estimation of how much is the list of genes enriched in a specific group of biological functions about what could occur by chance.

### 3.3 Semi-quantitative RT-PCR and quantitative RT-PCR analysis

Total RNA was copied to double strand DNA using the One-step reverse-transcription polymerase chain reaction (RT-PCR) kit (Qiagen) following the manufacturer instructions. For semi-quantitative analysis, PCR with specific primers was used to amplify the cDNA libraries made (17 cycles for U6, 27 cycles for *Trl* and *Skl*). Products were analysed on 1% Agarose gels. U6 was used as internal standard because it was taken as a bona fide gene not regulated by GAGA factor. Total RNA, without the reverse transcription step, was always used as a control for genomic DNA contamination.

Real-time quantitative PCR (qPCR) analysis from the cDNA libraries, prepared as described above, was performed using the Light Cycler system (Roche) and a Light Cycler 480 apparatus as described [25]. Primers used were the following:

GstE3 up_qPCR TTACTAGTCAATCGCCTTACAG
GstE3 low_qPCR GATCTTGTAGTCGAAGTCCA
GstE6 up_qPCR AGACGAAAGTACCCAAGGAG
GstE6 low_qPCR CGATGAAACGAGACTGAAATCC
GstE7 up_qPCR GAGAAGCATTACCAAGCCAC
GstE7 low_qPCR TAGACCTCAATAATCGCATCGT
SoxN up_qPCR CACGGAGAACCAACTTTGAG
SoxN low_qPCR CTGCCTTTCATATCCGATTCC
jhamt up_qPCR GTTTATGAAGGCGTGAGGAC
jhamt low_qPCR AGGAACTGTTCATGCAAATCTG
Skl up_qPCR CGCAACTTGTGATTACTTTACG
Skl low_qPCR CATTTCTCTGTCACTGTCTCG
SytIV up_qPCR CTTCGCATTCGATATTCCCG
SytIV low_qPCR ACCGATGACCTCATTCTTGG
Hsc70-1 up_qPCR GATGTCACTCCTCTGTCTCTG
Hsc70-1 low_qPCR AATCAGCGTAGTCATCACTCC
SK-RK up_qPCR TTTACATCCTGACACAGCTG
SK-RK low_qPCR CATCTCGATGTTAATCACGG
SK-RJ up_qPCR TGCACAACTTCATGATGGAC
SK-RJ low_qPCR CTTTGGTGGGTTCTTACACG
Rbp9 up_qPCR AGCGGACTATCTTTAATCCA
Rbp9 low_qPCR ATAATACTGCTGTGAAGTCCCT
mod(mdg4) up_qPCR TACAGTGAATCATCGTTTGTCG
mod(mdg4) low_qPCR GAACTTCTTGTGCAACAGCA

### 3.4 Antibodies and microscopy

Rabbit anti-GAGA antibody was obtained in the laboratory and used as described [19]. For immunostaining, rabbit anti-GFP (Molecular Probes) was used at 1 : 1000 dilution, rabbit anti-Skl (from E. Alnemri) was used at 1 : 600 dilution and mouse monoclonal anti-Ubx (FP3.38) (from R. White) were used at 1 : 10 dilution, rat anti-Mod(mdg4) (from V. Corces) at 1 : 600 dilution, rabbit anti-activated Caspase3 (Cell Signaling) at 1 : 100 dilution and rabbit anti-*γ*H2Av (Rockland) at 1 : 1000 dilution. Appropriate secondary antibodies coupled to Cy2, Cy3 or Cy5 (Jackson Immunochemicals) were used at 1 : 600 dilution.

Tissue immunostaining was performed as described [19]. Polytene chromosomes were prepared on slides by incubation of salivary glands in Cohen–Gotchell's solution (25 mM Na-Glycerol-3-phosphate, 10 mM KH_2_PO_4_ 30 mM KCl, 10 mM MgCl_2_, 3 mM CaCl_2_, 160 mM sucrose, 0.5% Nonidet P-40) for 8 min and fixed for 2 min in 2% formaldehyde in phosphate buffered saline (PBS), and 3 min in 45% acetic acid, 2% formaldehyde. Then, antibodies were added in blocking solution (PBS, 0.05% Tween 20, 1% bovine serum albumin (BSA)) and incubated overnight at 4°C in a moist chamber. Slides were washed three times in PBS containing 0.05% Tween 20 and incubated with fluorescently labelled secondary antibodies at 1 : 400 dilution in blocking solution. After three more washes in PBS containing 0.05% Tween 20 and one in PBS, preparations were mounted in DAPI-Mowiol solution.

All preparations were analysed using Leica confocal microscopes (SP2, SP5 and/or SPE) or Nikon E-800/E-1000 conventional fluorescent microscopes.

### 3.5 Protein extracts and western blot analysis

Total protein extracts were prepared from 20 to 40 wing discs grounded in SDS-PAGE protein loading buffer with a pestle. After 5 min at 95°C, debris were pellet by centrifugation. Supernatants were loaded on 10% SDS-PAGE gels and proteins were transferred to nitrocellulose membranes. Proteins were identified using rabbit anti-actin (Sigma) at 1 : 1000 dilution, mouse monoclonal anti-*β*tubulin (Millipore) at 1 : 10 000 dilution and rabbit anti-GAGA at 1 : 3000 dilution. Appropriate secondary horseradish peroxidase-conjugated antibodies (Jackson Immunochemicals or Amersham-G&E) were used at 1 : 10 000 dilution. Bands were visualized using enhanced chemiluminiscent detection systems (G&E Healthcare or Immobilon (Millipore)) and exposure to films (ThermoScientific). ImageJ (FIJI) was used for band quantification from films.

### 3.6 Other methods

Wings and legs were dissected, mounted in Faure's medium and pictures taken using a Nikon E600 microscope equipped with an Olympus DP72 camera as described [19]. Wing area analysis was performed separately for males and females using ImageJ with the pictures recorded (always *n*≥15). Whole fly pictures were taken on an Olympus SZX16 motorized scope equipped with an XC50 camera and Cell D software.

For cuticle preparation, embryos were treated with sodium hypochloryte to remove the corion, then washed briefly with 0.1% Triton X-100, and treated with methanol/heptane solution for 30 s (with vortexing). After two washes with methanol and one with 0.1% Triton X-100, embryos were spread on a microscope slide and covered with a drop of Hoyer's-lactic and a coverslide and incubated at 60°C overnight. Pictures were taken in a Nikon E1000 microscope using dark field conditions and a Cool-Snap fx camera.

GAGA-inducible Schneider S2 cells were cultured and immunostained as described [25].

## 4. Results

### 4.1 GAGA factor depletion reveals a limited set of genes changing expression in wing disc

GAGA distribution on the *Drosophila* genome is well known for embryos, S2 cells and, recently, even for 3rd instar imaginal wing discs. However, there is limited information about genome-wide GAGA effects on gene expression. With the aim to shed some light on GAGA contribution at larval stages, transcriptome analysis was carried out in 3rd instar larval wing discs. A combined approach using GAGA knockdown and highly restricted GAGA overexpression conditions was used.

**Figure 1.**
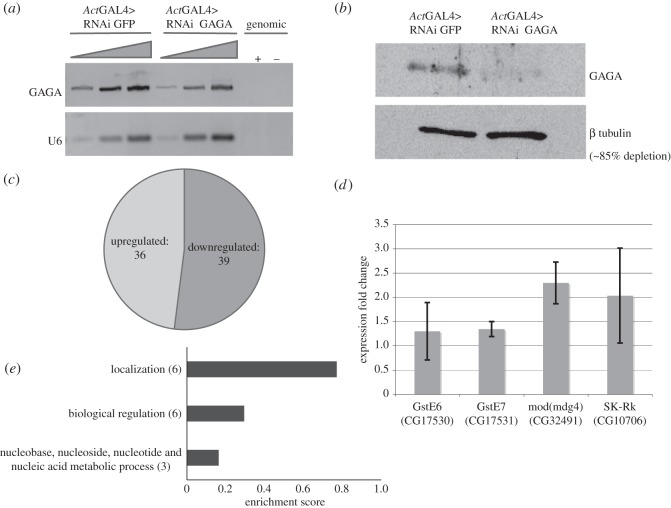
Analysis of the results obtained by microarray analysis of 3rd instar wing disc GAGA depletion. (*a*) RT-PCR of total RNA extracted from discs depleted of GAGA using RNAi GAGA and discs depleted of an irrelevant RNAi (RNAi GFP) used as a control. Upper panel indicates GAGA mRNA, lower panel indicates U6 snRNA and was used as internal control. The rightmost two lanes show the signal of the assay for the same RNA samples without RT and indicate the absence of genomic DNA contamination. (*b*) Western blot of wing discs either depleted of GAGA or mock-depleted (RNAi GFP) as control. Upper panel indicates GAGA protein levels and lower panel indicates *β* tubulin levels of the same gel used as a loading internal control. (*c*) Distribution of upregulated genes (light grey) and downregulated genes (dark grey) obtained from the analysis of the microarray data of GAGA-depleted discs. (*d*) RT-qPCR analysis of some genes selected from the list of genes that changed expression in the microarray analysis. Results are shown as the mean of three independent experiments. Error bars indicate s.e.m. (*e*) Cluster analysis of the upregulated genes according to their DAVID enrichment score. In brackets the number of genes for each cluster is indicated.

Depletion of GAGA factor using RNAi and tissue-specific drivers usually showed mild effects in flies although some phenotypes were reported [19]. Nevertheless, a rather general GAGA depletion using *Act*GAL4 drivers (in chromosomes II and III) could not be used because it was embryonic lethal in any combination with all GAGA RNAi strains (not shown). A good GAGA depletion, that was not affecting viability, was obtained by using 69BGAL4 driver that is expressed widely in the wing disc at 25°C. At 29°C, it caused strong lethality at pupa stage (not shown). Time restriction in 69BGAL4-mediated RNAi expression, by using the *tub*GAL80^ts^, resulted in insufficient GAGA depletion and was not used (not shown). Using 69BGAL4 UAS GAGA RNAi at 25°C, GAGA factor expression was remarkably reduced (approx. 85%, figure 1*a*,*b*) and allowed microarray analysis of the wing disc transcriptome. In parallel, experiments were run in which the expression of an irrelevant RNAi (GFP RNAi) was used as a control for non-specific effects due to the activation of the RNAi machinery and GAL4 overexpression. After subtraction of the GFP RNAi control, the results obtained showed that only 75 genes changed their expression (fold change≥2.0), 39 were downregulated, and 36 were upregulated (figure 1*c*; electronic supplementary material, table S1 that lists all results obtained in the microarray analysis for GAGA depletion). Using the recently published ChipSeq data for GAGA factor in wing discs [26], we determined that 19 of the downregulated genes (i.e. 54%) and 14 of the upregulated genes (i.e. 39%) presented GAGA factor in wild-type conditions (electronic supplementary material, table S1). For these calculations non-assigned probe sets (NA; electronic supplementary material, table S1) were not taken into consideration.

Because of the extensive RNAi treatment more changes were expected. However, even when the microarray analysis used less stringent conditions similar profiles were obtained (not shown). Moreover, indirect effects may account for the similar number of upregulated and downregulated genes observed and, to some extent, they were expected. Noteworthy, none of the genes previously reported to be under GAGA factor regulation in embryos, *Ubx, en, ftz, eve, Adh, act5C*, etc., showed any change in 3rd instar larvae. RT-qPCR analysis validated some of these results (*GstE7*, *mod(mdg4), SK-Rk*, figure 1*d*). Cluster analysis for upregulated genes highlighted in the microarray analysis, using the Functional Annotation Clustering tool of DAVID package, showed a moderate enrichment for genes involved in localization and biological regulation, and a lower enrichment for genes involved metabolism of nucleotides, nucleosides and nucleic acids metabolism. The same analysis did not provide any cluster for the downregulated genes, although a significant group of six genes on oxidation/reduction showed a *p*-value of 3.6299×10^−4^ using the Functional Annotation Chart tool of the DAVID package (figure 1*e* and results not shown). These results were surprising because GAGA factor being a transactivator, we expected the downregulated genes to be confirmed upon depletion. In addition, because of the many GAGA sites shown by the genome-wide mapping in wing discs, a larger amount of genes were expected to be under GAGA regulation [26]. These results together with the strong effects shown by null mutations in embryos and by depletion using *Act*GAL4 (not shown) suggested that GAGA contribution was largely different in embryos than in larvae.

### 4.2 GAGA factor depletion revealed several phenotypes mostly in the adult

**Figure 2.**
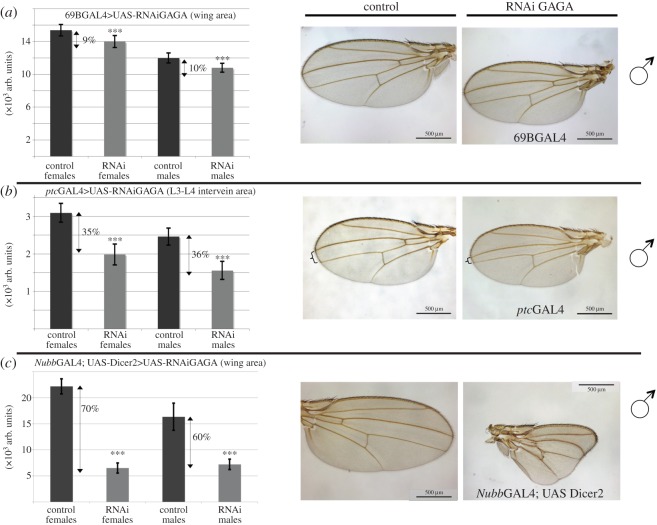
GAGA depletion affects cell proliferation in wings. (*a*) GAGA depletion using 69BGAL4. Left panel shows quantification of the whole wing area for control and depleted females and males, respectively (*p*-values for Student's *t*-test were 3.86×10^−14^ and 5.22×10^−13^ for females and males, respectively; *n*>30 in all cases). (*b*) GAGA depletion using *ptc*GAL4. Left panel shows quantification of the wing area between L3 and L4 veins (corresponding to the *ptc* expression domain in wings, indicated by keys in the pictures) for control and depleted females and males, respectively (*p*-values for Student's *t*-test were 1.02×10^−35^ and 4.12×10^−19^ for females and males, respectively; *n*>35 for all cases, except for RNAi females where *n*=16). (*c*) GAGA depletion using *Nubb*GAL4; UASDicer2. Left panel shows quantification of the whole wing area for control and depleted females and males, respectively (*p*-values for Student's *t*-test were 9.14×10^−61^ and 3.75×10^−37^ for females and males, respectively; *n*>50 for all RNAi samples, *n*>20 for controls). For all panels, numbers indicate the relative decrease of wing area separately for males and females. Central and right panels show images of control and interfered male wings, respectively. Female images were similar in all cases (not shown).

Taking into account the depletion achieved in these experiments (approx. 85%; figure 1*b*) it was somehow surprising that only few genes changed expression and that none of them were previously reported in the analysis of GAGA loss of function experiments. Previous genetic experiments correlated the appearance of phenotypes in the adult to the gene of interest but only occasionally a phenotype was described in imaginal tissues. In the conditions used for our experiments, several abnormalities were detected also in the adult. Thus, GAGA depletion using 69B resulted in a moderate but consistent wing size reduction (approx. 10%; figure 2*a*). This abnormality was further confirmed and enhanced using two other GAL4 drivers whose expression appeared stronger than 69B (*ptc*GAL4 approx. 35%, and *Nubb*GAL4 in combination with Dicer2 overexpression >55%; figure 2*b*,*c*). Because tricome density was similar in all cases, it could be concluded that the reduction in wing size was due to a defect in cell proliferation.

**Figure 3.**
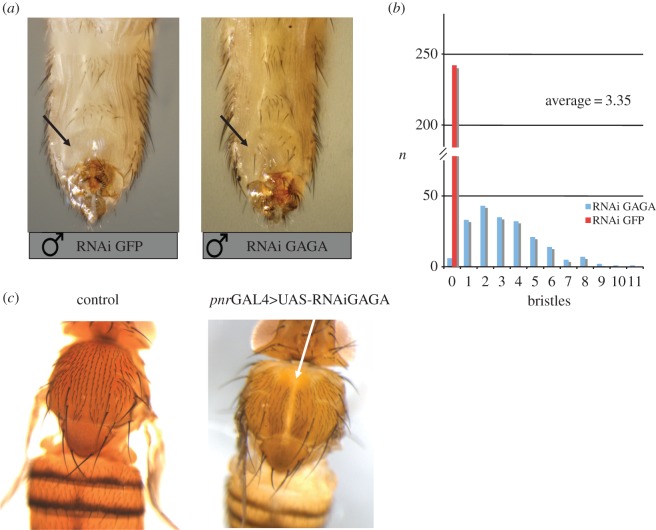
GAGA depletion impairs expression in the adult. (*a*) Abdominal images from mock-depleted (using 69BGAL4>RNAi GFP, on the left) and GAGA-depleted (using 69BGAL4>RNAi GAGA, on the right) males. Arrow indicates the absence of bristles in the sixth abdominal segment (A6) in the mock-depleted males and the presence of several bristles in the same segment of GAGA-depleted males. (*b*) Quantification of the presence of bristles on A6 and its distribution in GAGA-depleted (*n*=200, blue bars) and in mock-depleted (*n*=243, red bar) male flies. (*c*) Adult dorsal images from control (left) and GAGA-depleted (right) flies (using *pnr*GAL4). White arrow on the right panel shows the dorsal closure phenotype.

Similarly, abdominal segment A6 showed a homeotic transformation to A5 as revealed by the appearance of bristles in the A6 segment in males (figure 3) upon GAGA factor depletion with 69B (figure 3*a*). A detailed analysis of the number of bristles showed that it was rather high in comparison with the corresponding control (3.35 bristles per A6 segment on the average versus 0 in the 69B mediated GFP_RNAi_ depletion as control, *p*<1.06×10^−7^; figure 3*b*). These results were indicative of homeotic transformation of abdominal segment A6 to A5 and were attributed to a defect in *Abd-B* expression. A similar homeotic transformation was previously observed in heterozygous males carrying a null *Trl* allele in front of a deficiency (*Trl*^67^/*Df*(*3R*)*Sbd26*) [27]. The same result was obtained using independent GAGA RNAi strains and other GAL4 drivers ([28], and results not shown).

Another phenotype observed after GAGA depletion using *pannier*GAL4 (*pnr*GAL4) was a defect in dorsal closure as shown by a cleft deprived of bristles on the dorsal part of notum (figure 3*c*). Remarkably, a much stronger phenotype affecting dorsal closure was observed after GAGA overexpression using the same driver. In this case, embryos died before reaching larval 1st instar stage and displayed a clear defect in dorsal closure usually manifested by the presence of a rather big hole (electronic supplementary material, figure S1 shows the embryonic phenotype of GAGA overexpression using *pnr*GAL4).

On the other hand, and contrary to the published [1], no significant alteration of UBX expression was detected in our experiments by phenotype analysis, even in a *Ubx*^130^ sensitized background. Despite a considerable reduction in GAGA content, imaginal disc immunostaining could only show a slight reduction of UBX in the best case (electronic supplementary material, figure S2 shows UBX immunostainings of GAGA-depleted imaginal discs, and results not shown).

**Figure 4.**
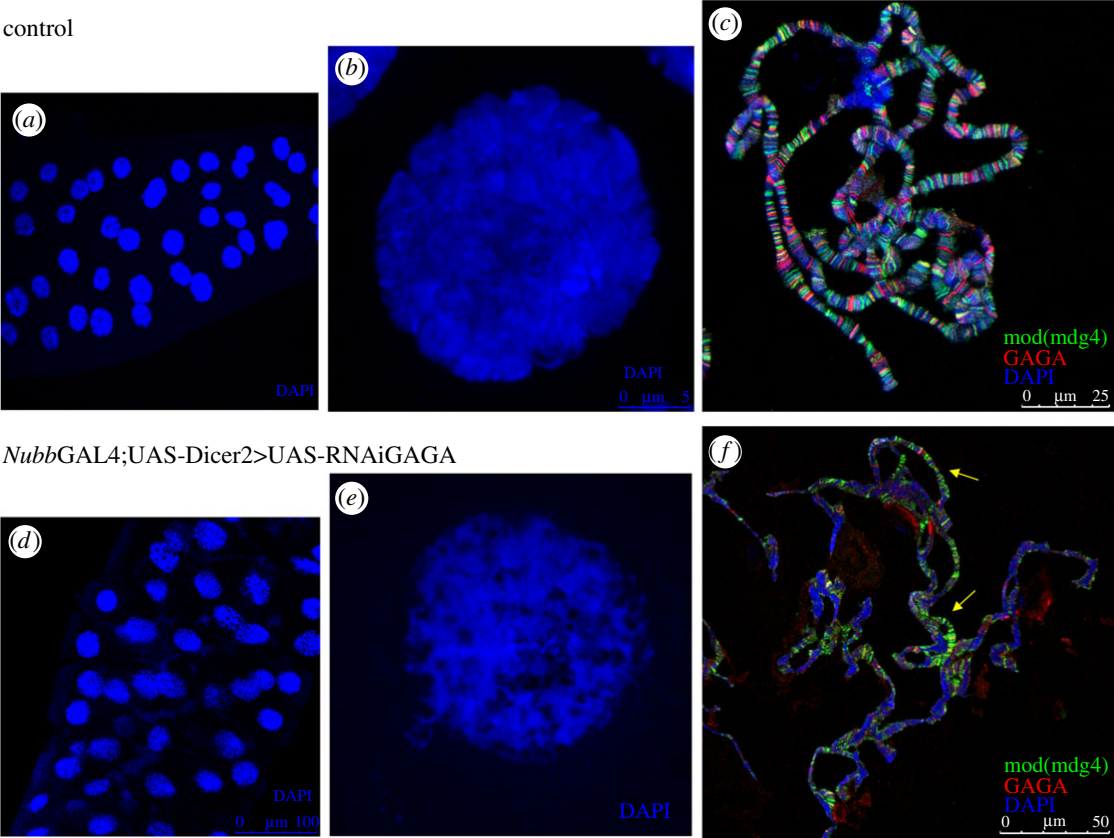
GAGA depletion affects chromosome organization. (*a*) Confocal image of control salivary glands. DNA was stained with DAPI. (*b*) High magnification of a nucleus from (*a*) shows normal polytene chromosome organization. (*c*) Immunostained polytene chromosome preparation from control salivary glands shows a normal distribution for mod(mdg4) (in green), GAGA factor (in red) and DAPI staining indicates DNA (in blue). (*d*) Confocal image of GAGA-depleted salivary glands. DNA was stained with DAPI. (*e*) High magnification of a nucleus from (*d*) shows abnormal polytene chromosome organization. (*f*) Immunostained polytene chromosome preparation from GAGA-depleted salivary glands shows highly distorted polytene chromosomes and abnormal distribution for mod(mdg4) (in green) and GAGA factor (highly reduced, in red). DAPI staining indicates DNA (in blue). Arrows indicate regions in which polytene chromosome structure is less distorted than in the rest.

While we were studying GAGA contribution in 3rd instar wing discs a strong phenotype was observed in salivary glands. Depletion of GAGA factor using *Nubb*GAL4 in combination with enhanced expression of Dicer2 resulted in apparently normal salivary glands but DNA staining showed a very abnormal nuclear structure in which DNA in blue appeared not in the usual polytene chromosome structure but as thin filaments around many ‘black holes’ giving the image of a ‘sponge-like’ nuclear structure (figure 4 cf. *a* and *d*, and *b* and *e* (at higher magnification), respectively). When polytene chromosome spreads were prepared from these glands aberrations became evident (figure 4 cf. panels *c* and *f*). Chromosome alignment was disturbed and regions thinner than normal were frequently observed. This polytene chromosome phenotype resembled that obtained on histone H1 depletion although salivary gland nuclear structure did not [29–31]. Because *mod*(*mdg4*) showed an altered expression in the microarray experiments described above and taking into account its role in chromatin organization, its distribution on these polytene chromosomes was analysed. Mod(mdg4) staining of GAGA-depleted polytene chromosomes revealed such a strong rearrangement that any defined region was difficult to recognize. GAGA factor staining was clearly fainter than in controls, as expected. Note, however, that some small regions still retained some staining (figure 4*f*, arrows). In fact, this picture was obtained from crosses kept at 18°C and reflected one of the mildest effects observed. Frequently, when prepared from crosses at 25°C, chromosomes appeared much more disturbed, heavily fragmented, and GAGA and Mod(mdg4) staining were further reduced (not shown). These results indicated that GAGA factor depletion strongly altered polytene chromosome organization in salivary glands. Nevertheless, these polytene chromosome aberrations did not affect pupation and flies hatched normally showing only the wing defects described above. These effects on chromosome structure were reminiscent to those described in early embryos from mutant mothers that included failure in chromosome condensation, abnormal chromosome segregation, and chromosome fractionation in addition to asynchrony in the cleavage cycles [17]. Although we have no direct data indicative of these aberrations in imaginal wing disc cells, we noted that DNA damage occurred on GAGA depletion as revealed by the appearance of *γ*H2Av spots highly concentrated in the wing pouch and nearly absent outside when *Nubb*GAL4; UASDicer2 was used for the depletion experiments (electronic supplementary material, figure S3 shows anti *γ*H2Av immunostainings of wing discs depleted of GAGA factor).

### 4.3 GAGA factor overexpression also reveals a limited set of different genes changing expression in wing disc

**Figure 5.**
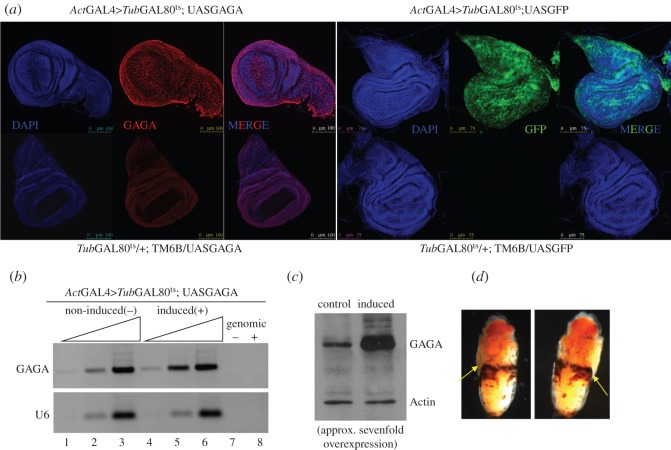
Characterization of GAGA overexpression in wing discs. (*a*) Immunostaining of wing discs reveals overexpression of GAGA (upper left panels, in red) and GFP (upper right panels, in green) after 13 h at 29°C using *act*GAL4. Lower left and right panels show their respective controls from the same crosses. DNA was stained with DAPI (in blue). (*b*) RT-PCR of total RNA extracted from discs overexpressing GAGA (induced) and control (non-induced) as before. Upper panel indicates GAGA mRNA, lower panel indicates U6 snRNA used as internal control. The rightmost two lanes show the signal of the assay for the same RNA samples without RT and indicate the absence of genomic DNA contamination. (*c*) Western blot of wing discs either overexpressing GAGA or controls as above. Actin was used as an internal control. (*d*) Two examples of the phenotype presented by pupae after GAGA overexpression for 13 h at 29°C at 3rd instar larvae and subsequently brought back to 18°C.

A complementary set of experiments using GAGA overexpression was carried out. Owing to the high lethality observed on GAGA overexpression [19], the *tub*GAL80^ts^ UAS-GAL4 system was used. With this set-up, expression of the GAGA factor was kept repressed at 18°C because GAL80 interacts with GAL4 activation domain and renders it unable to activate transcription [32]. At the desired time (3rd instar larval stage), flies were shifted to 29°C to inactivate GAL80 and to allow robust expression of GAGA factor mediated by GAL4. To minimize indirect effects, induction was limited to 13–16 h. Free wandering larvae were then collected and wing discs processed for analysis. Using *Act*GAL4 as a driver, a strong GAGA overexpression was obtained without apparently affecting wing disc morphology (figure 5*a*). An increase of approximately 1.8- to 2.0-fold in mRNA levels (as determined using ImageJ analysis, cf. tracks 1–2 and 4–5 in figure 5*b*) and approximately sevenfold in protein levels was obtained (figure 5*c*). In these conditions no larval lethality was observed but this short overexpression was sufficient to cause pupae lethality (even if after 13 h at 29°C the temperature was shifted back to 18°C, not shown). All pupae overexpressing GAGA factor died and displayed a dark necrotic ring in a central position (figure 5*d*) while the flies carrying the balancing chromosome showed no lethality (TM6B, as indicated by the associated tubby phenotype, not shown).

**Figure 6.**
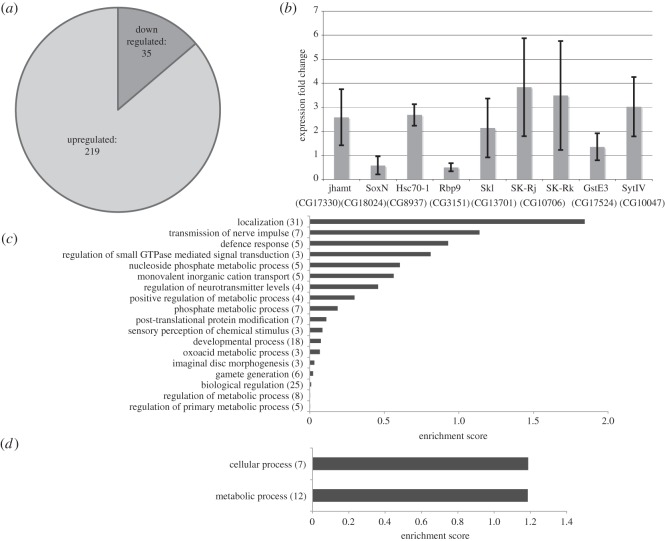
Analysis of the results obtained by microarray analysis of 3rd instar wing disc GAGA overexpression for 13 h at 29°C. (*a*) Distribution of upregulated (light grey) and downregulated genes (dark grey). (*b*) qRT-PCR analysis of several genes selected from the list of genes that changed expression in the microarray analysis. Results are shown as the mean of three independent experiments. Error bars indicate s.e.m. (*c*) Cluster analysis of the upregulated genes according to their DAVID enrichment score. (*d*) Cluster analysis of the downregulated genes according to their DAVID enrichment score. In brackets, the number of genes for each cluster is indicated.

Microarray analysis of mRNAs compared the amounts of mRNAs between samples after induction of GAGA or GFP expression (GFP was used as a control gene to correct for changes in expression owing to the temperature shift). Two hundred and nineteen genes showed increased expression and 35 genes showed decreased expression (fold change≥2.0) after subtraction of the corresponding GFP overexpression control (figure 6*a*; electronic supplementary material, table S2 that shows microarray results for GAGA overexpression experiments in wing disc). Most of the changes due to GAGA overexpression reflected activation as expected for the overexpression of a transactivator. Quantitative real-time PCR analysis validated these results for some selected target genes. Among the upregulated genes, *Hsc70-1*, *Skl*,*SK* (Rj and Rk transcripts), *GstE3, Rbp9* and *SytIV* were selected either as representative of the more frequent positives (three probes for *Hsc70-1*, two probes for *SK*) or for its positive regulation in a similar microarray analysis performed in S2 cells (*Skl, GstE3*) [25], or randomly. Among the potentially downregulated genes, *jhamt* and *SoxN* were selected. Results of three independent experiments confirmed the changes in the expression of many of them (figure 6*b*) with the exception of *jhamt* and *Rbp9*. This discrepancy might be due to their very low expression levels in 3rd instar discs according to Graveley *et al.* [18].

Using recently published ChipSeq data for GAGA factor in wing discs [26], we determined that among the genes listed in the electronic supplementary material, table S2, that 67 of the upregulated genes presented GAGA factor bound (i.e. 37% of genes), and only one gene, SoxN, of the downregulated genes in wild-type conditions (i.e. 14%). For these calculations non-assigned probe sets (NA; electronic supplementary material, table S2 that shows microarray results for GAGA overexpression experiments in wing disc) were not taken into account.

Cluster analysis for the upregulated genes using the Functional Annotation Clustering tool of DAVID package, revealed an enrichment of genes involved in localization, transmission of nerve impulse, defence response and regulation of small GTPase-mediated signal transduction, among other less relevant clusters (figure 6*c*). Also, an enrichment of genes for cellular and metabolic processes was observed for the downregulated genes (figure 6*d*). Remarkable in this analysis were the insignificant enrichment of the cluster for developmental process and the absence of any regulation of transcription cluster (see below). Phenotype analysis in the adult was not possible in these conditions because of lethality at pupa described above. Nevertheless, GAGA overexpression resulted in salivary glands smaller in size than control glands. Since cell number did not change significantly, this reduction in size was due to a cell size smaller than in the control. Nuclei also appeared slightly smaller and chromosomes looked more densely packed than in the control (electronic supplementary material, figure S4 shows salivary glands from control and GAGA overexpressing larvae), adding evidence that GAGA levels affected chromosome organization.

**Figure 7.**
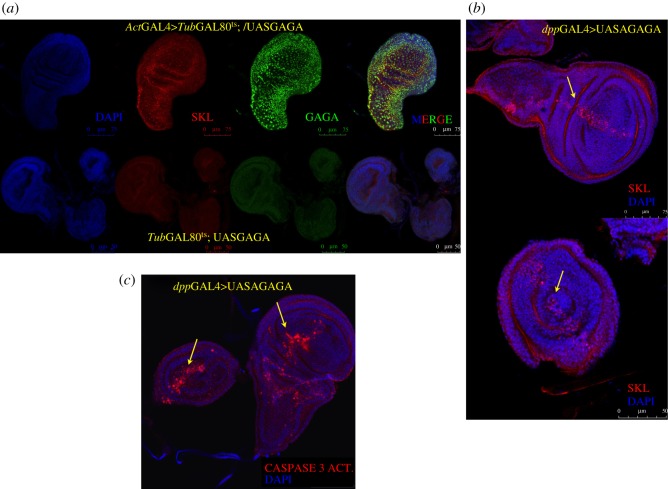
The proapototic gene *Skl* (Sickle) is ectopically expressed upon GAGA overexpression. (*a*) Immunostaining of wing discs reveals overexpression of GAGA (green) and SKL (red) after 13 h at 29°C using *act*GAL4 (upper panels). DNA was stained with DAPI (in blue). Lower panels show controls from the same crosses. (*b*) Immunostaining of wing and leg discs overexpressing GAGA factor using *dpp*GAL4 shows ectopic expression of SKL around the antero/posterior axis (AP) (in red). DNA was stained with DAPI (in blue). (*c*) Immunostaining of wing and leg discs overexpressing GAGA factor using *dpp*GAL4 shows activated Caspase 3 around AP axis (in red). DNA was stained with DAPI (in blue).

The upregulation of Sickle, *Skl*, after GAGA overexpression called our attention because it also appeared upregulated in a similar analysis previously performed in S2 cells [25]. *Skl* is a proapoptotic gene whose overexpression might help to explain some of the phenotypes observed [33,34]. Immunostaining of wing imaginal discs with anti-SKL specific antibodies confirmed the ectopic expression of SKL protein in larvae overexpressing GAGA factor in the same conditions as used for the microarray experiments (figure 7*a*). At the RNA level, semi-quantitative RT-PCR analysis showed that, while *Skl* mRNA was almost undetectable before induction, it was easily detected after induction (electronic supplementary material, figure S5 shows semi-quantitative RT-PCR analysis of Skl expression in wing discs overexpressing GAGA factor) confirming real-time qPCR results obtained (figure 6*b*). Because general overexpression of GAGA factor using *tub*GAL80^ts^; *Act*GAL4 was limited to 13–16 h, immunostaining of SKL in the wing disc showed only a faint overexpression of SKL. To confirm these results, GAGA was overexpressed in wing discs using *dpp*GAL4 to a well-defined narrow area around the anterior/posterior axis, leaving the rest of the disc as a negative control. In these conditions GAGA overexpression at 25°C was not lethal at larval stages. Specific immunostaining clearly showed an upregulation of *Skl* in the expected region when compared with the rest of the disc (figure 7*b*). Furthermore, this activation of *Skl* expression resulted in apoptosis as revealed by the specific staining of the same region using anti-Caspase 3 activated antibodies (figure 7*c*). Notably, no signs of *Skl* upregulation were observed in embryos and other larval tissues after GAGA overexpression, suggesting that *Skl* upregulation was context-dependent (not shown). The upregulation of *Skl* after GAGA factor overexpression was also confirmed in S2 cells (electronic supplementary material, figure S6*a*). Because all these results suggested *Skl* as a good candidate for a direct target of GAGA factor, the *Skl* proximal promoter region (approx. 600 bp upstream from transcription start site) was cloned and tested in transient transfection experiments in S2 cells. The *Skl* proximal promoter region showed a clear GAGA factor dose-dependent activation in transient transfection experiments (electronic supplementary material, figure S6*b* shows that *Skl* expression is dose-dependent activated by GAGA in transiently transfected S2 cells) suggesting that GAGA alone might directly activate the *Skl* promoter in S2 cells and potentially in imaginal discs.

## 5. Discussion

*Drosophila* GAGA factor is a multifunctional regulator of gene expression that activates transcription, promotes chromatin remodelling [11,35,36] and also acts in gene silencing by acting on a limited number of PREs, usually in combination with Dorsal Switch Protein 1 (DSP1) and zeste, facilitating the access of Polycomb complexes [10,13,16,37–39]. In fact, Polycomb Repressive Complex 1 (PRC1) members transiently co-immunoprecipitate with GAGA factor in two different complexes in embryos [40]. On the other hand, a central activity of GAGA factor in the concentration of TFs at highly occupied target regions (HOT) has been recently shown [41].

Most of these roles have been defined in embryos, and little is known about GAGA function(s) at later stages. Here, we have studied GAGA factor in 3rd instar larvae wing discs using overexpression and depletion experiments. In contrast to the several thousand binding sites described for GAGA factor in the fly genome [15,16,26], our results showed a limited list of genes that changed expression either by overexpression or by depletion. Overlap between the two conditions was minimal and, with high confidence (*p*-value=7.244×10^−8^), no correlation could be established (Spearman corr.=0.039; good correlation should give values close to 1.0, not shown). Nevertheless, for the few genes appearing in both assays (only six), changes notably went in the opposite direction (five upregulated for overexpression and downregulated for depletion, and only one downregulated for overexpression and upregulated for depletion). In addition, none of the genes that changed expression was previously reported as regulated by GAGA, and many of them have unknown functions. Unfortunately, the lack of any obvious connection between the significantly enriched clusters obtained and the phenotypes observed in cluster analysis did not provide any explanation to the phenotypes observed. While, conceivably, depletion could have been insufficient to reveal some targets, several phenotypic effects were described, arguing in favour of a reasonable depletion was achieved. On the other hand, overexpression experiments can be prone to false positives, in general, because of a number of reasons. In our experiments we have been especially accurate to minimize this point. In fact, at least for one gene (*Skl*) that has been studied in detail, this seems unlikely because of its dose-dependent activation in S2 cells and its lack of upregulation in tissues other than imaginal discs (e.g. embryos, not shown). Even in this case, apoptosis was shown not to be enough for explaining the phenotype observed because coexpression of the anti-apoptotic gene DIAP1 could not abolish the phenotype observed but only alleviate it, resembling the results obtained when GAGA overexpression was not allowed before 1st instar larval stage (electronic supplementary material, figure S7 shows several phenotypes observed in adults upon time-controlled GAGA overexpression).

GAGA binding sites are reported for embryos, for the *Drosophila* S2 cell line, and, recently for wing discs [15,16,26]. Because a large fraction of sites in the wing discs are the same as in embryos and S2 cells our results in the wing disc are suggesting different roles for GAGA factor in the two cases. While GAGA is reported to control several genes involved in the embryo development, in the wing disc it seems to control a different set of genes, mainly housekeeping genes. GAGA was previously reported to regulate some housekeeping genes (e.g. actin5C, alcohol dehydrogenase, *α*-1-tubulin) [4,20]. However, as a member of the Trithorax group, it was expected to keep open and active the chromatin of certain homeotic genes too. Microarray analysis did not indicate any change in the expression of homeotic genes on GAGA dosage. *Ubx* is a homeotic gene whose expression was reported to depend on GAGA factor [1]. Since *Ubx* is not expressed in wing discs, but in haltere discs, it was not expected to show any effect on GAGA dosage. However, the effects of GAGA depletion on *Ubx* were expected to be visible as a haltere to wing homeotic transformation in the adults. Despite many efforts, and using conditions that showed good depletion in the haltere disc (*Nubb*GAL4; UASDicer2, see electronic supplementary material, figure S2), we could not observe any visible defects of *Ubx* expression in adults largely depleted of GAGA in haltere discs even in a sensitized *Ubx*^130^ hypomorph background (not shown). To reconcile these different results we suggest that either this phenotype is set up at very early stages in embryo development (before RNAi could efficiently deplete GAGA factor to the required level) or that it is indirect. GAGA loss-of-function clonal analysis reached similar conclusions [42] and recent results confirmed that GAGA regulation of *Ubx* expression is, at least, uncertain [43]. As a difference, GAGA depletion experiments showed homeotic transformation of abdominal segment A6 into A5 (figure 3), indicating that *Abd-B* expression was altered. The same result was previously observed using *Trl* mutants either in homozygosis or in combination with a deficiency [1,27]. Together, these results indicate that GAGA depletion at 3rd instar larval stage was comparable to that achieved using *Trl* mutants and, indirectly, give support to the microarray results obtained in wing discs and discarding an insufficient depletion of GAGA factor as the cause for the lack of phenotypes.

Another line of evidence that confirmed previous results was the effect on the reduction of wing size after GAGA depletion (figure 2). We could not observe any significant size reduction in other organs, like salivary glands, imaginal discs, legs, halteres or the whole body either using several GAL4 vectors (results not shown). In the wing, these results suggested an effect on growth that was consistent with the presence of less cells per surface unit as shown by a similar trichome density. GAGA has recently been shown to be required for the correct activity of the *dE2f1*-*Yorkie*(*Yki*)/*Scalloped*(*Sd*) programme that directs proper tissue growth [44]. Indeed, GAGA factor has been found to interact directly with Yki in complex and chromatin binding studies, and also supported an extensive functional overlap between Yki and GAGA, indicating that they are frequent partners for the transcriptional regulation of downstream genes. In fact, activated Yki required GAGA, Brahma and mediator [26]. However, none of the target genes of this pathway was highlighted in our wing disc microarray analysis, or in the previous microarray analysis in S2 cells (see the electronic supplementary material, tables S1 and S2 for microarray lists of upregulated and downregulated genes and [25]). Moreover, in S2 cells, GAGA factor depletion did not affect cell growth (D. Piñeyro and J. Bernués 2010, unpublished data). We do not know the reasons for that but we suspect that many of the changes observed at the adult stage could reflect that, while set-up during larval stages, they only become functionally relevant by the combination with additional factors during pupal stages when massive rearrangement, apoptosis, and proliferation take place [45].

The dramatic effects of GAGA depletion on polytene chromosomes, and the detection of DNA damage in wing disc cells, suggest that similar chromosomal abnormalities might also take place in diploid cells. These results may underlie a new potential role for GAGA factor on chromosome structure and organization. Although indirect effects cannot be discarded, GAGA factor interaction with mod(mdg4) was shown to bypass insulator function and, more in general, the involvement of GAGA factor in the insulator/boundary elements revealed an effect on the 3D organization of chromatin [5,14,46,47]. Also, GAGA was reported to bring two DNA molecules close together to activate enhancers in trans [48]. These and other results [41] suggest that GAGA factor could be acting in the establishment of the appropriate genomic architecture for the correct expression of genes and suggest GAGA as one of the TFs that might coordinate gene expression. Of note, this regulation is not due to a reduced GAGA factor amount in larvae. In fact, GAGA mRNA expression is high in 3rd instar wing discs according to data in FlyBase (flybase.org/reports/FBgn0013263.html) and GAGA factor protein expression is quite constant during *Drosophila* development despite GAGA mRNA levels fluctuations (results not shown).

In *Drosophila*, HOT sites are >5000 genomic regions characterized by the binding of more than 14 TFs. GAGA factor is highly enriched and constitutive at HOT sites where GAGA binding motifs are abundant, as a difference to most TFs that appear to bind them indirectly because of the absence of their respective binding sequences [49]. Therefore, a large fraction of GAGA factor may play an important role in maintaining them or influencing their regulatory output through interaction with NELF and its association with paused RNA polymerase II [15,50,51]. Also, by its association with regions of low-nucleosome occupancy and by its interaction with NURF, FACT and PABP complexes [47,52–54], GAGA may allow binding of the other TFs most likely by protein-protein interactions. Notably, among the thousands of HOT sites present in the *Drosophila* genome, only a part of them have been found to be active enhancers, usually regulating genes involved in development, and those were shown to be dependent on the binding of additional TFs elsewhere [49]. Transcriptome analysis during the *Drosophila* life cycle has shown that at the embryo-to-larva transition there is an abrupt change in transcription in which highly transcribed embryonic genes, mostly involved in development and regulation of gene expression, become downregulated or off, whereas a new set of genes, mainly housekeeping genes involved in metabolism, become upregulated. At the larva-to-pupa transition genes change similarly but in the other direction and many of the genes highly transcribed at embryo become upregulated again [55]. Thus, despite the continuous binding of GAGA factor to a large majority of its target genes during the whole *Drosophila* life cycle, our results suggest that GAGA factor is changing the genes that regulate at 3rd instar wing discs with respect to embryos, a change in regulation that recapitulates the global change in gene expression observed at these two stages. Thus, GAGA depletion could not affect many HOT regions in the larvae probably because they are mainly silent at this time.

Transcriptional regulation of a gene is frequently (if not always) part of a transcription network that is highly connected to other transcription networks in the cell nucleus to make transcriptional regulation an integrated continuum [56]. In this sense, we think GAGA is a good example in the light of the accumulated literature and the recent description of its involvement in the nucleation of many active enhancers at HOT regions [41,49].

## Supplementary Material

Supplementary Figures 1 to 7

## Supplementary Material

Supplementary Tables 1 and 2

## Supplementary Material

Text for Suppl. Figures 1 to 7

## Data accessibility

Microarray experiments data available from the Dryad Digital Repository: http://dx.doi.org/10.5061/dryad.kp328.
