# Supplementary material for: New insights for Drosophila GAGA factor in larvae
Source: R Soc Open Sci. 2015 Mar 18;2(3):150011. doi: 10.1098/rsos.150011 (PMC4448821; doi:10.1098/rsos.150011)
Supplement: Supplementary Figures 1 to 7 [file rsos150011supp1.ppt]

## Slide 1
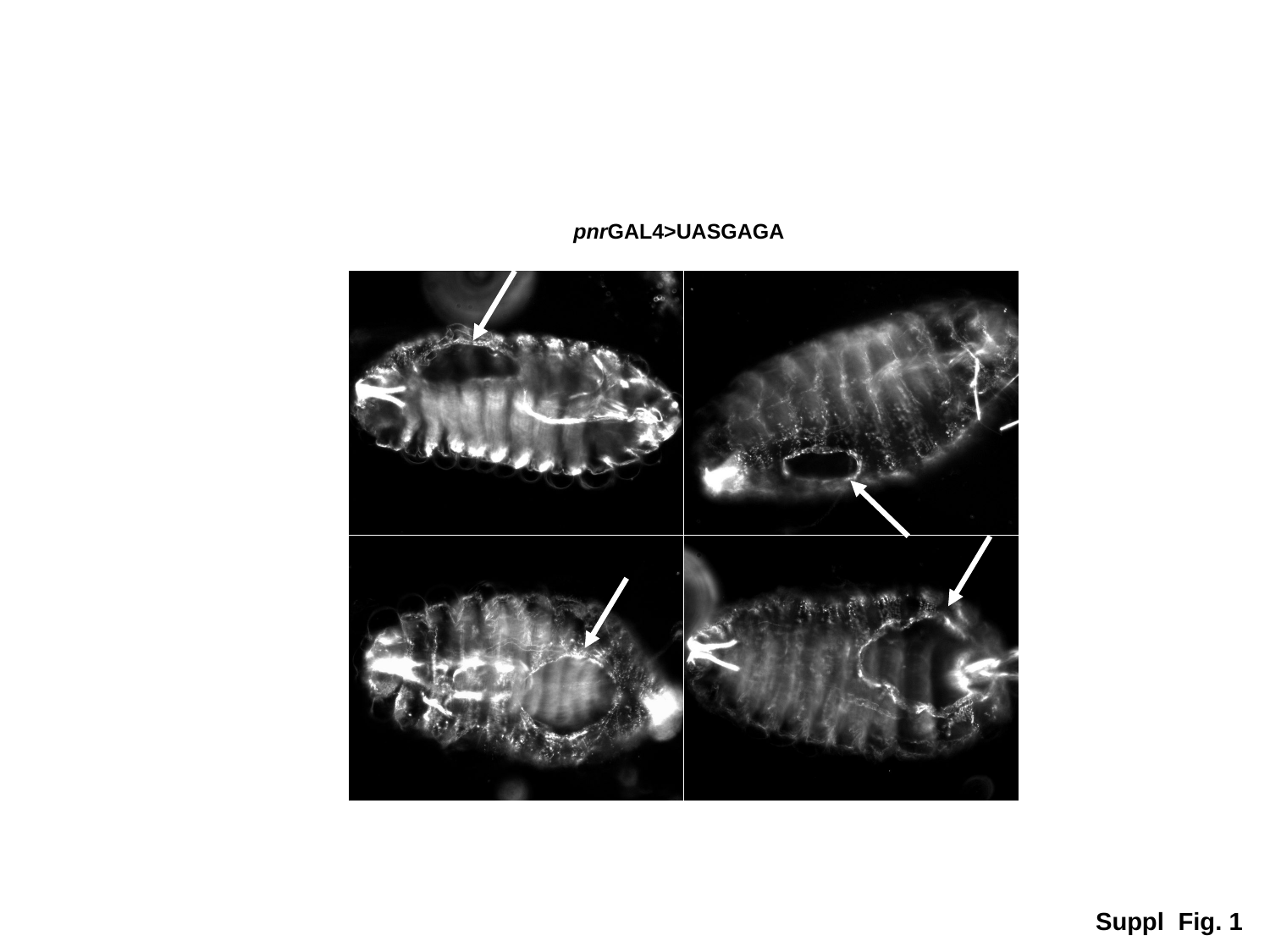

pnrGAL4>UASGAGA
Suppl Fig. 1

## Slide 2
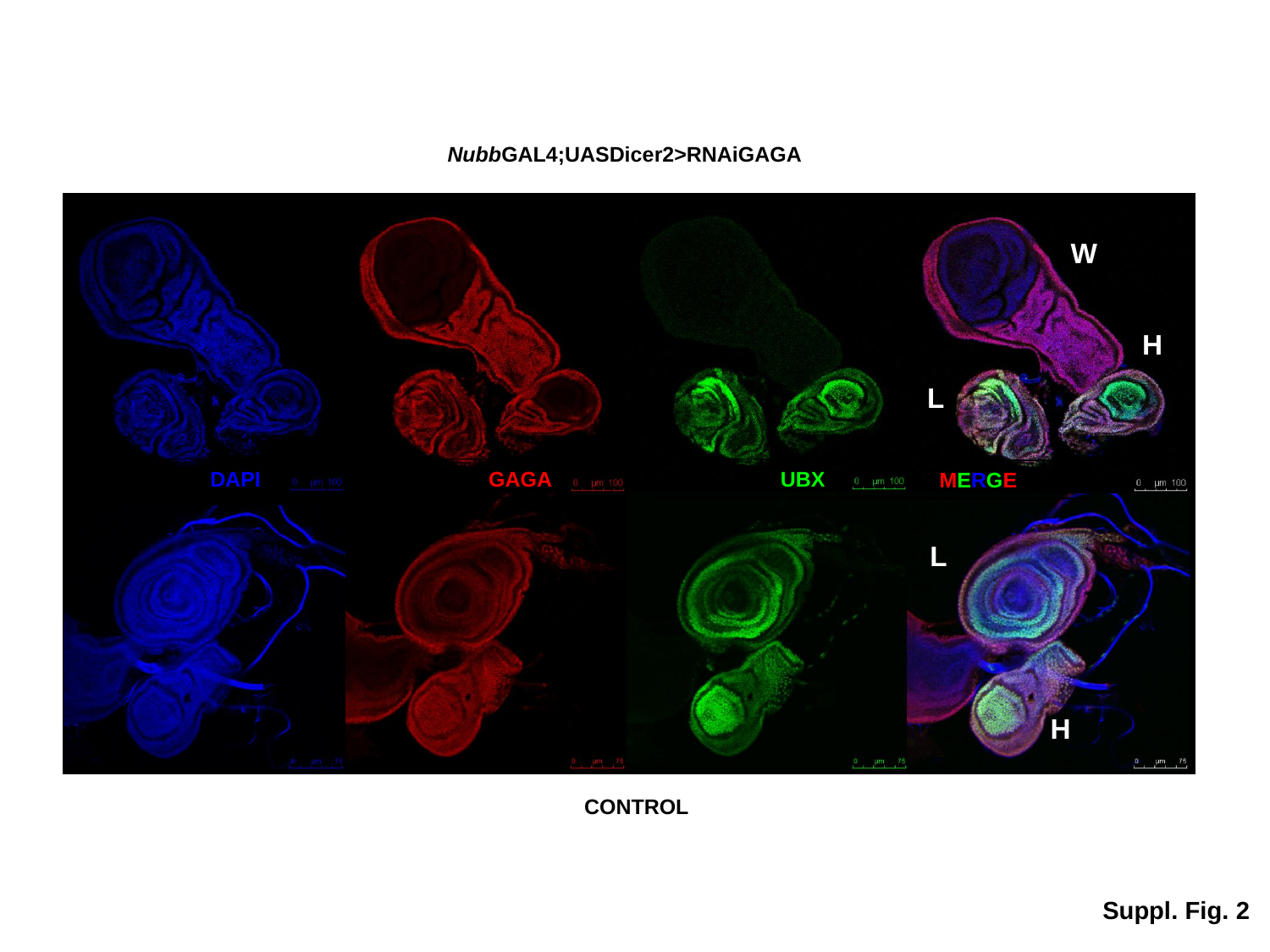

NubbGAL4;UASDicer2>RNAiGAGA
W
H
L
DAPI
GAGA
UBX
MERGE
L
H
CONTROL
Suppl. Fig. 2

## Slide 3
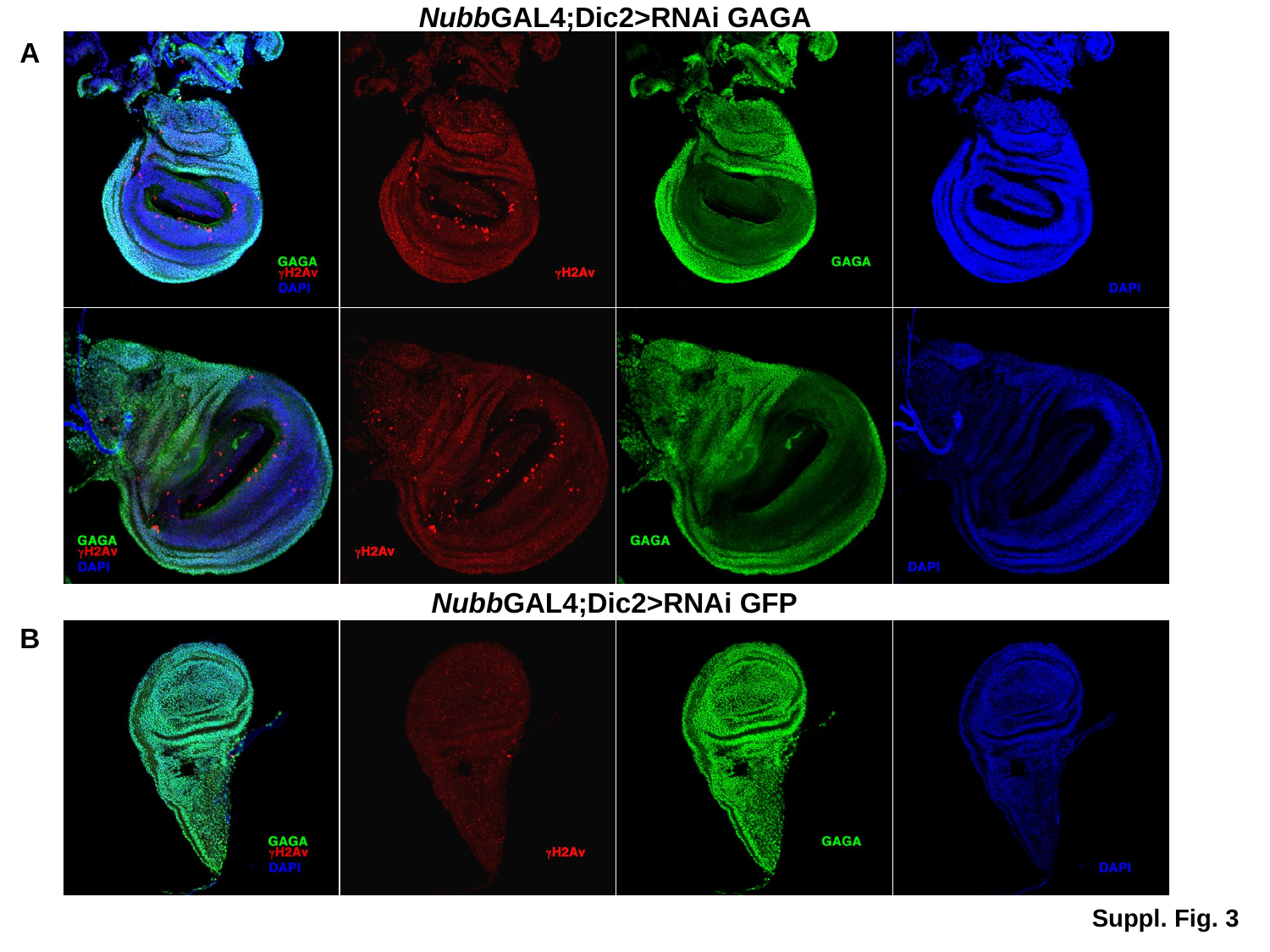

NubbGAL4;Dic2>RNAi GAGA
NubbGAL4;Dic2>RNAi GFP
A
B
Suppl. Fig. 3

## Slide 4
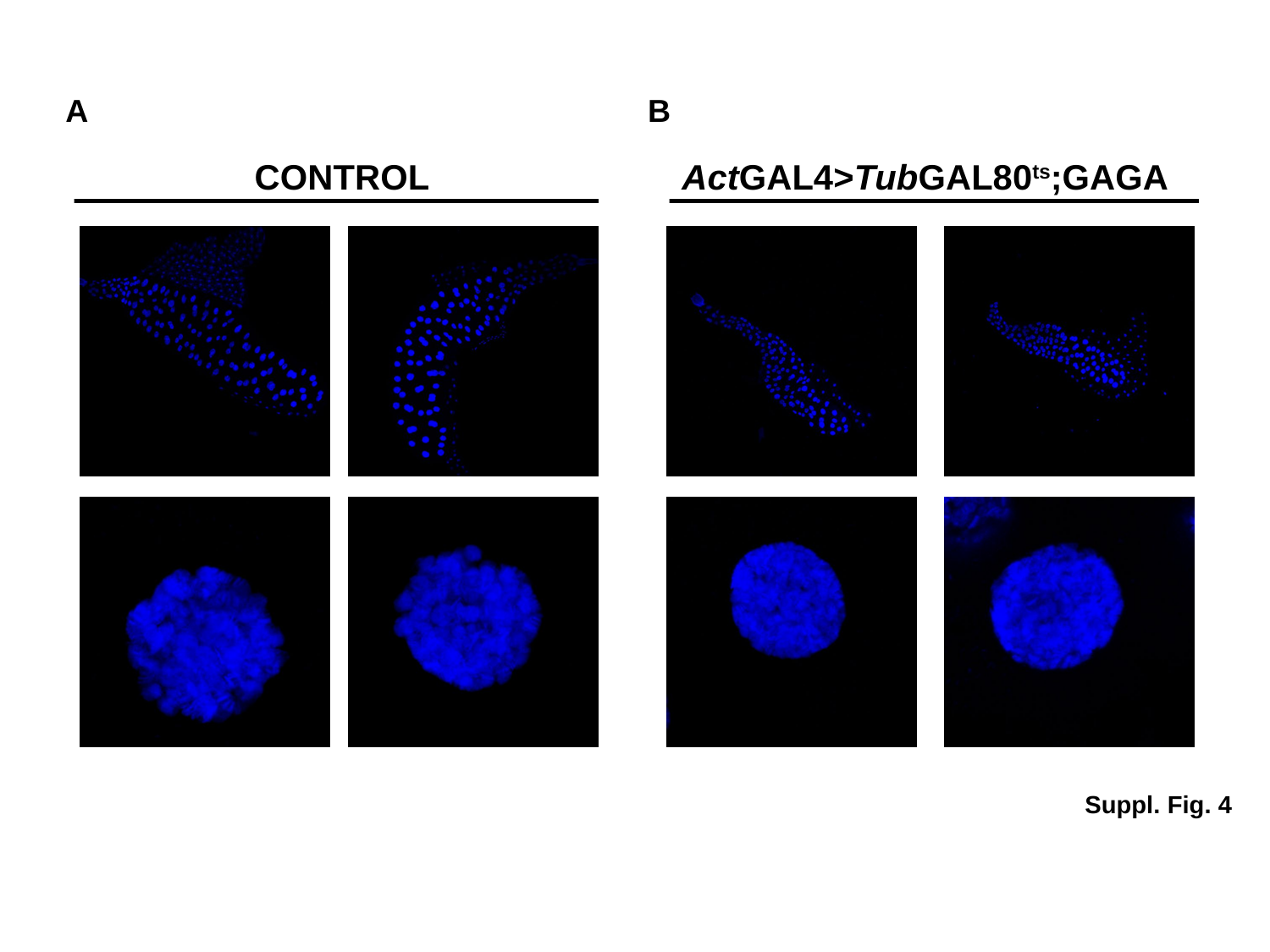

A
B
CONTROL
ActGAL4>TubGAL80ts;GAGA
Suppl. Fig. 4

## Slide 5
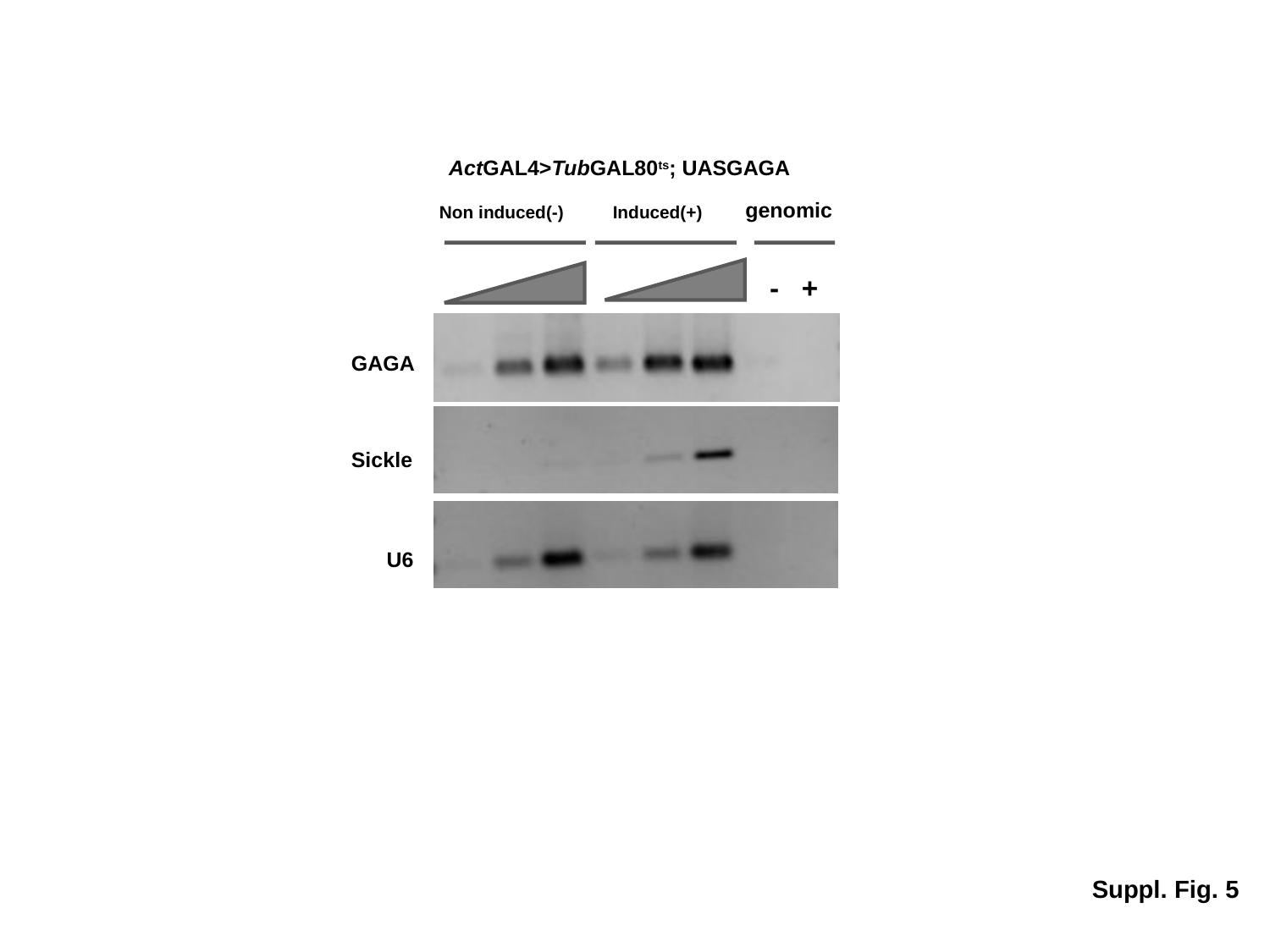

ActGAL4>TubGAL80ts; UASGAGA
genomic
Non induced(-)
Induced(+)
 -
 +
GAGA
Sickle
U6
Suppl. Fig. 5

## Slide 6
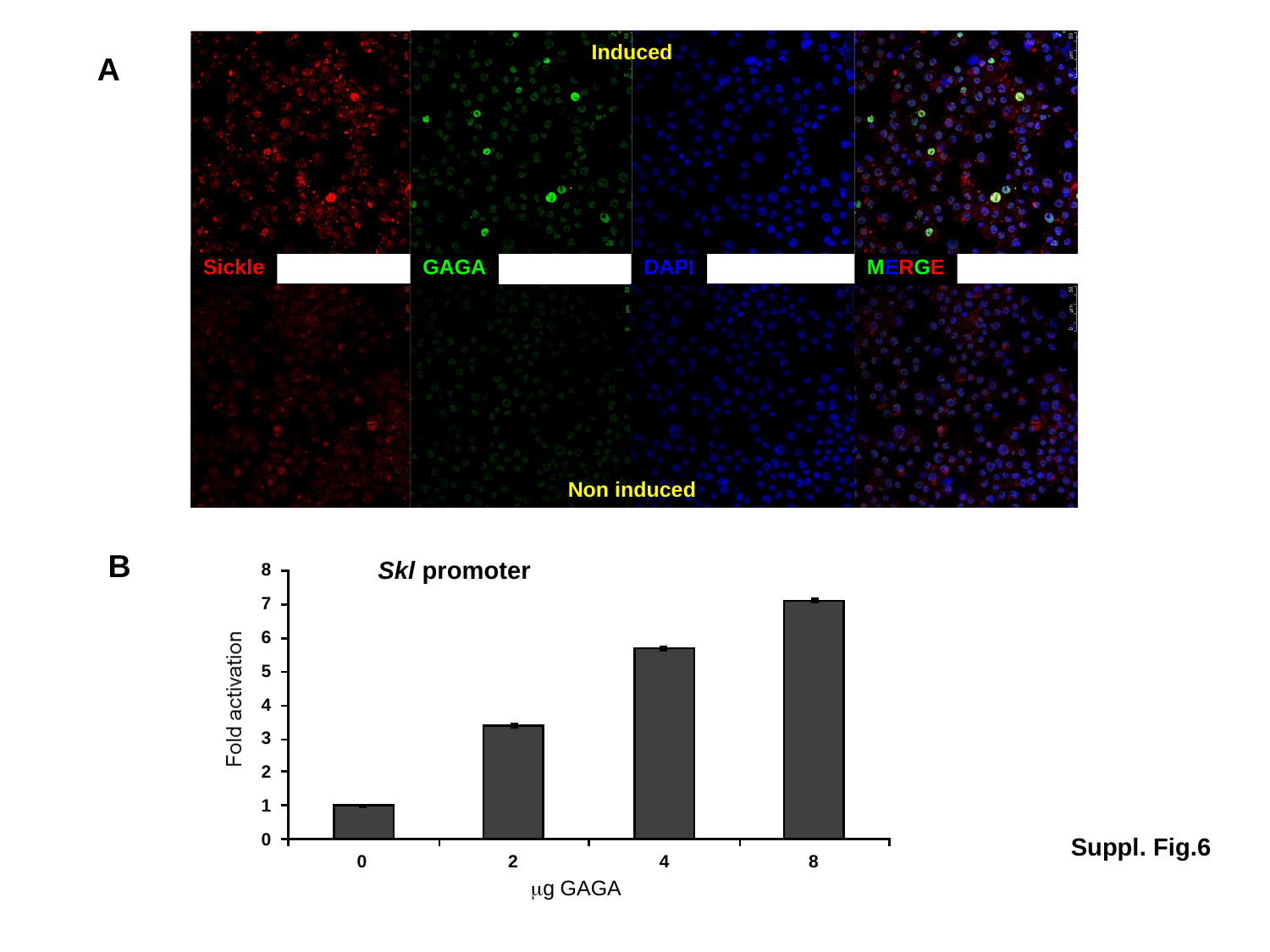

Induced
A
MERGE
Sickle
GAGA
DAPI
Non induced
Skl promoter
μg GAGA
B
Suppl. Fig.6

## Slide 7
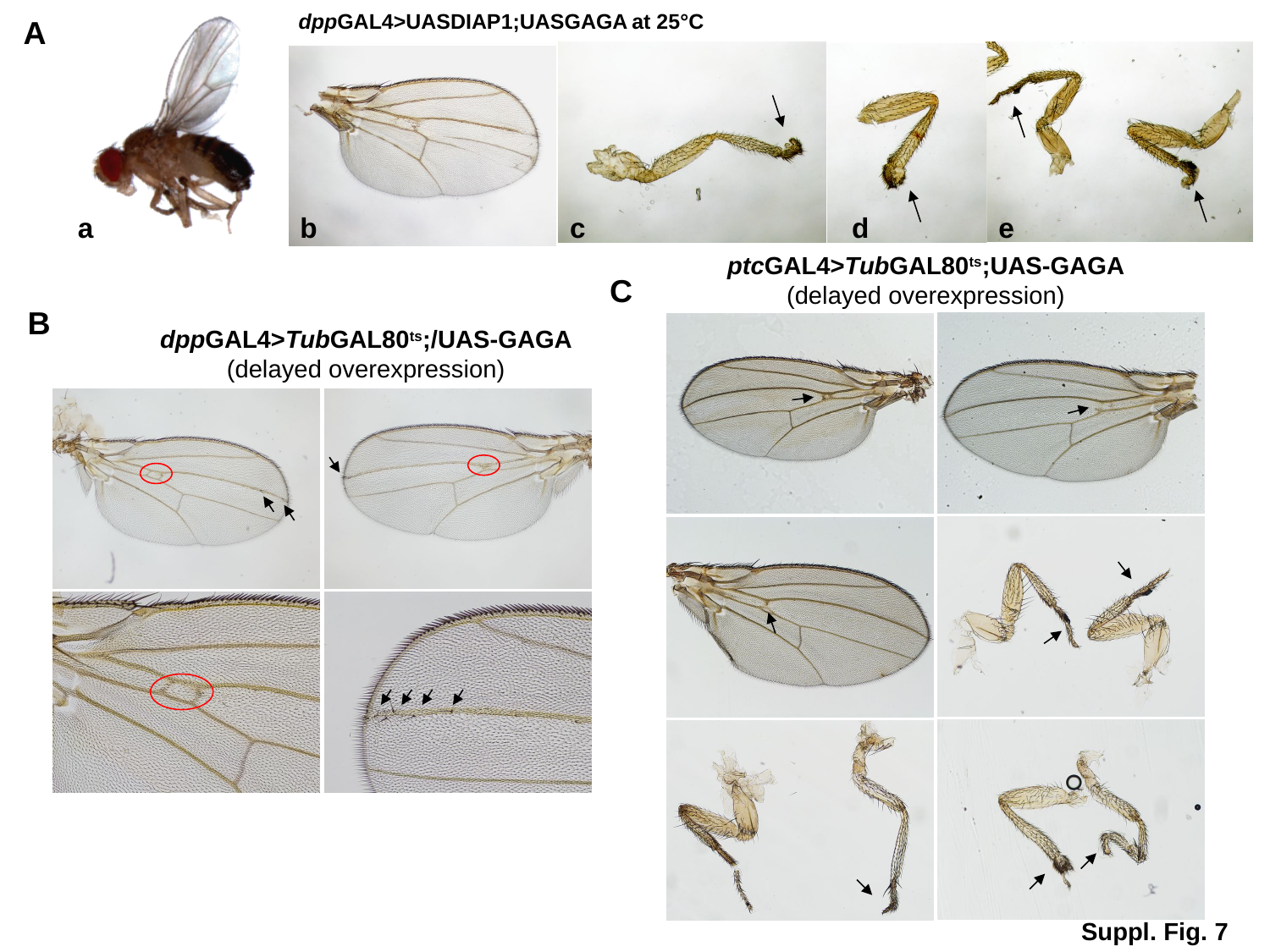

dppGAL4>UASDIAP1;UASGAGA at 25°C
A
a
b
c
d
e
ptcGAL4>TubGAL80ts;UAS-GAGA
(delayed overexpression)
C
B
dppGAL4>TubGAL80ts;/UAS-GAGA
(delayed overexpression)
Suppl. Fig. 7
