# Supplementary material for: New insights for Drosophila GAGA factor in larvae
Source: R Soc Open Sci. 2015 Mar 18;2(3):150011. doi: 10.1098/rsos.150011 (PMC4448821; doi:10.1098/rsos.150011)
Supplement: Supplementary Tables 1 and 2 [file rsos150011supp2.doc]

Supplemental Tables

Table S1

**Genes that change expression after GAGA depletion**

**Downregulated genes**

| symbol | | genename | | fc | ChipSeq [26] |  |
| --- | --- | --- | --- | --- | --- | --- |
| CG12972 | CG12972 gene product from transcript CG12972-RA | | -2,094523483 | | + | |
| CG18335 | CG18335 gene product from transcript CG18335-RA | | -2,099127169 | | + | |
| CG12481 | CG12481 gene product from transcript CG12481-RB | | -2,112140157 | | - | |
| NA | NA | | -2,147191562 | |  | |
| Cyp4e2 | Cytochrome P450-4e2 | | -2,176116843 | | + | |
| CG30488 | CG30488 gene product from transcript CG30488-RA | | -2,207287888 | | - | |
| Ugt86Di | CG6658 gene product from transcript CG6658-RA | | -2,271143559 | | + | |
| CG7916 | CG7916 gene product from transcript CG7916-RA | | -2,278452224 | | - | |
| CG2201 | CG2201 gene product from transcript CG2201-RA | | -2,332756607 | | + | |
| Pu | Punch | | -2,386215854 | | + | |
| GstE6 | Glutathione S transferase E6 | | -2,487950639 | | + | |
| CG31988 | CG31988 gene product from transcript CG31988-RA | | -2,497141056 | | - | |
| Cyp6d4 | CG12800 gene product from transcript CG12800-RA | | -2,519621033 | | + | |
| CG9150 | CG9150 gene product from transcript CG9150-RA | | -2,525098152 | | - | |
| CG7328 | CG7328 gene product from transcript CG7328-RA | | -2,539359743 | | - | |
| Cyp6d2 | CG4373 gene product from transcript CG4373-RA | | -2,651771676 | | - | |
| Oseg5 | CG9333 gene product from transcript CG9333-RB | | -2,722720778 | | - | |
| CG8800 | CG8800 gene product from transcript CG8800-RA | | -2,755419123 | | - | |
| CG30195 | CG30195 gene product from transcript CG30195-RA | | -2,836503374 | | + | |
| CG6678 | CG6678 gene product from transcript CG6678-RA | | -2,949638683 | | - | |
| NA | NA | | -3,01291492 | |  | |
| GstE7 | Glutathione S transferase E7 | | -3,040030116 | | + | |
| CanA-14F | Calcineurin A at 14F | | -3,070833783 | | + | |
| Zip3 | Zinc/iron regulated transporter-related protein 3 | | -3,076435139 | | + | |
| CG12842 | CG12842 gene product from transcript CG12842-RA | | -3,184414193 | | + | |
| CG13045 | CG13045 gene product from transcript CG13045-RA | | -3,423954161 | | + | |
| CG30154 | CG30154 gene product from transcript CG30154-RA | | -3,553063772 | | - | |
| CG33144 | CG33144 gene product from transcript CG33144-RA | | -3,725950015 | | + | |
| CG7768 | CG7768 gene product from transcript CG7768-RB | | -3,830928622 | | + | |
| NA | NA | | -4,605847037 | |  | |
| NA | NA | | -4,782756193 | |  | |
| CG32572 | CG32572 gene product from transcript CG32572-RA | | -6,455772905 | | + | |
| Tsp68C | Tetraspanin 68C | | -6,729539206 | | - | |
| NA | NA | | -7,592173116 | |  | |
| CG32368 | CG32368 gene product from transcript CG32368-RA | | -8,34881599 | | + | |
| unc-13-4A | CG32381 gene product from transcript CG32381-RB | | -9,932715661 | | - | |
| CG32850 | CG32850 gene product from transcript CG32850-RA | | -22,26248874 | | - | |
| CG15236 | CG15236 gene product from transcript CG15236-RB | | -31,35194639 | | + | |
| Cyp4p1 | Cytochrome P450-4p1 | | -33,46369939 | | - | |
| CG2177 | CG2177 gene product from transcript CG2177-RA | | -45,11306431 | | - | |

**Upregulated genes**

| symbol | genename | fc | ChipSeq [26] |
| --- | --- | --- | --- |
| SytIV | Synaptotagmin IV | 66,87746816 | - |
| gp210 | CG7897 gene product from transcript CG7897-RA | 34,22531957 | + |
| Tektin-C | Tektin C | 31,17742936 | - |
| Caps | Calcium activated protein for secretion | 12,44189574 | + |
| Caps | Calcium activated protein for secretion | 9,58725836 | + |
| CG5217 | CG5217 gene product from transcript CG5217-RA | 7,494049933 | - |
| CG15270 | CG15270 gene product from transcript CG15270-RA | 6,07673556 | + |
| NA | NA | 5,971943183 |  |
| CG1139 | CG1139 gene product from transcript CG1139-RA | 5,932554394 | - |
| CG7900 | CG7900 gene product from transcript CG7900-RB | 5,318903107 | + |
| CG6330 | CG6330 gene product from transcript CG6330-RB | 4,404424552 | + |
| Pxd | Peroxidase | 3,771890062 | + |
| mod(mdg4) | modifier of mdg4 | 3,686538908 | + |
| CG5281 | CG5281 gene product from transcript CG5281-RA | 3,610426473 | + |
| NA | NA | 3,541287296 |  |
| NA | NA | 3,388781409 |  |
| Cpr47Eb | Cuticular protein 47Eb | 3,37673252 | - |
| Ac76E | Adenylyl cyclase 76E | 3,358721214 | + |
| CG2241 | CG2241 gene product from transcript CG2241-RA | 2,888491589 | - |
| eIF4E-3 | CG8023 gene product from transcript CG8023-RA | 2,853009603 | - |
| CG32027 | CG32027 gene product from transcript CG32027-RA | 2,779254657 | + |
| CG3253 | CG3253 gene product from transcript CG3253-RA | 2,720764092 | - |
| CG10570 | CG10570 gene product from transcript CG10570-RA | 2,692957806 | - |
| CG34104 | CG34104 gene product from transcript CG34104-RB | 2,440642733 | + |
| NA | NA | 2,336979468 |  |
| yellow-h | CG1629 gene product from transcript CG1629-RA | 2,298801205 | - |
| CG13707 | CG13707 gene product from transcript CG13707-RA | 2,280544469 | - |
| Fili | Fish-lips | 2,236509297 | + |
| NA | NA | 2,231191562 |  |
| dpr19 | CG13140 gene product from transcript CG13140-RA | 2,214680762 | - |
| CG31755 | CG31755 gene product from transcript CG31755-RA | 2,211229818 | - |
| CG32313 | CG32313 gene product from transcript CG32313-RA | 2,184846571 | + |
| CG13258 | CG13258 gene product from transcript CG13258-RA | 2,163195988 | - |
| NA | NA | 2,14061753 |  |
| CG33469 | CG33469 gene product from transcript CG33469-RA | 2,034584329 | - |
| CG4562 | CG4562 gene product from transcript CG4562-RB | 2,028589318 | + |
| CG10232 | CG10232 gene product from transcript CG10232-RA | 2,008213926 | - |

Genes that change expression on GAGA depletion ordered according to their fold change values. Only those with a fold change ≥ 2.0 are listed.

Table S2

**Genes that change expression after GAGA overexpression**

Upregulated genes

| symbol | genename | fc | Chipseq[26] |
| --- | --- | --- | --- |
| CG13215 | CG13215 gene product from transcript CG13215-RA | 75,97819832 | - |
| Ntl | Neurotransmitter transporter-like | 33,95576208 | - |
| CG14661 | CG14661 gene product from transcript CG14661-RA | 26,29042536 | + |
| Syt1 | Synaptotagmin 1 | 20,3540929 | - |
| qua | quail | 19,74065546 | - |
| Ank2 | CG34416 gene product from transcript CG34416-RK | 19,59851529 | - |
| Muc14A | Mucin 14A | 18,23081381 | - |
| Adgf-A | Adenosine deaminase-related growth factor A | 17,4716873 | - |
| Cpr49Ae | Cuticular protein 49Ae | 16,60717715 | - |
| Hsc70-1 | Heat shock protein cognate 1 | 15,5509246 | - |
| Cyp6d2 | CG4373 gene product from transcript CG4373-RA | 14,3629756 | - |
| CG2150 | CG2150 gene product from transcript CG2150-RA | 14,07429561 | + |
| Cbp53E | Calbindin 53E | 13,72876352 | - |
| CG14227 | CG14227 gene product from transcript CG14227-RB | 13,69124076 | + |
| NA | NA | 13,11876845 |  |
| Ace | Acetylcholine esterase | 13,04180292 | + |
| CG31150 | CG31150 gene product from transcript CG31150-RA | 12,696936 | - |
| hig | hikaru genki | 12,45142681 | + |
| Ela | Elastin-like | 12,36535596 | - |
| Hsc70-1 | Heat shock protein cognate 1 | 12,36405775 | - |
| CG15145 | CG15145 gene product from transcript CG15145-RA | 11,77096406 | - |
| CG13822 | CG13822 gene product from transcript CG13822-RA | 11,3196758 | - |
| NA | NA | 11,31335576 |  |
| CG7886 | CG7886 gene product from transcript CG7886-RA | 11,0651376 | - |
| SK | small conductance Ca^2+^-activated potassium channel | 10,80343707 | + |
| skl | sickle | 10,387127 | + |
| SK | small conductance Ca^2+^-activated potassium channel | 10,11476528 | + |
| GluClalpha | CG7535 gene product from transcript CG7535-RC | 9,772136232 | + |
| CG12239 | CG12239 gene product from transcript CG12239-RA | 9,336128758 | - |
| CG8539 | CG8539 gene product from transcript CG8539-RA | 9,323644677 | - |
| Myo28B1 | CG6976 gene product from transcript CG6976-RB | 9,245714756 | - |
| CG6765 | CG6765 gene product from transcript CG6765-RA | 9,2035915 | + |
| NA | NA | 9,109443896 |  |
| CG14107 | CG14107 gene product from transcript CG14107-RA | 9,077120508 | - |
| neuroligin | CG13772 gene product from transcript CG13772-RA | 8,320452708 | - |
| CG2065 | CG2065 gene product from transcript CG2065-RA | 8,291088289 | + |
| CG13067 | CG13067 gene product from transcript CG13067-RA | 8,043744548 | - |
| Takl2 | Tak1-like 2 | 7,858807864 | + |
| CG30196 | CG30196 gene product from transcript CG30196-RA | 7,703544209 | - |
| dei | delilah | 7,537099017 | + |
| CG4815 | CG4815 gene product from transcript CG4815-RA | 7,482325417 | + |
| CG31976 | CG31976 gene product from transcript CG31976-RB | 7,453806877 | - |
| CG32191 | CG32191 gene product from transcript CG32191-RA | 7,295611165 | - |
| Eaat2 | Excitatory amino acid transporter 2 | 7,186271142 | - |
| CG8738 | CG8738 gene product from transcript CG8738-RA | 7,166451819 | - |
| clumsy | CG8681 gene product from transcript CG8681-RB | 7,02887534 | - |
| Ndg | Nidogen/entactin | 6,85728699 | - |
| Dhc36C | Dynein heavy chain at 36C | 6,743638459 | - |
| CG34283 | CG34283 gene product from transcript CG34283-RA | 6,610064217 | - |
| CG15556 | CG15556 gene product from transcript CG15556-RA | 6,545377082 | - |
| GluClalpha | CG7535 gene product from transcript CG7535-RC | 6,435340445 | + |
| CG14186 | CG14186 gene product from transcript CG14186-RA | 6,370788977 | + |
| Tsp42Ek | Tetraspanin 42Ek | 6,340286275 | - |
| Tsp68C | Tetraspanin 68C | 6,194898206 | - |
| CG30104 | CG30104 gene product from transcript CG30104-RB | 6,1904461 | - |
| CG15394 | CG15394 gene product from transcript CG15394-RB | 6,126055617 | - |
| Syt4 | Synaptotagmin 4 | 6,042161873 | - |
| Sdic1 | Sperm-specific dynein intermediate chain 1 | 6,019563539 | + |
| l(2)01289 | lethal (2) 01289 | 5,98874336 | - |
| NA | NA | 5,971078375 |  |
| CG10433 | CG10433 gene product from transcript CG10433-RA | 5,875424966 | + |
| CG17321 | CG17321 gene product from transcript CG17321-RA | 5,851247008 | + |
| CG42322 | CG42322 gene product from transcript CG42322-RI | 5,834870533 | + |
| CG32458 | CG32458 gene product from transcript CG32458-RA | 5,824900416 | - |
| CG4288 | CG4288 gene product from transcript CG4288-RB | 5,807764749 | - |
| Rbp9 | RNA-binding protein 9 | 5,807068225 | + |
| CG6675 | CG6675 gene product from transcript CG6675-RB | 5,799227146 | + |
| CG9717 | CG9717 gene product from transcript CG9717-RA | 5,782393243 | + |
| CG10512 | CG10512 gene product from transcript CG10512-RA | 5,763478411 | + |
| CG9813 | CG9813 gene product from transcript CG9813-RE | 5,712649528 | + |
| Cyp4e1 | Cytochrome P450-4e1 | 5,680285745 | + |
| CG30049 | CG30049 gene product from transcript CG30049-RA | 5,626452417 | - |
| CG31674 | CG31674 gene product from transcript CG31674-RA | 5,514161898 | - |
| Cyp28a5 | CG8864 gene product from transcript CG8864-RA | 5,441725895 | - |
| CG4267 | CG4267 gene product from transcript CG4267-RA | 5,367532475 | + |
| Obp44a | CG2297 gene product from transcript CG2297-RA | 5,327960224 | - |
| CG31103 | CG31103 gene product from transcript CG31103-RB | 5,326076735 | - |
| NA | NA | 5,279936648 |  |
| CG30195 | CG30195 gene product from transcript CG30195-RA | 5,197739522 | + |
| CG15917 | CG15917 gene product from transcript CG15917-RA | 5,176521134 | - |
| CG31619 | CG31619 gene product from transcript CG31619-RC | 5,107104699 | + |
| BicC | Bicaudal C | 5,088714273 | - |
| CG31686 | CG31686 gene product from transcript CG31686-RA | 5,046084342 | - |
| Lcp65Ag3 | Larval cuticle protein | 4,916598152 | - |
| ndl | nudel | 4,863280426 | - |
| CG8736 | CG8736 gene product from transcript CG8736-RB | 4,84194484 | - |
| CG9826 | CG9826 gene product from transcript CG9826-RA | 4,802796687 | - |
| CG18557 | CG18557 gene product from transcript CG18557-RA | 4,727864273 | - |
| BobA | Brother of Bearded A | 4,708335594 | - |
| CG2861 | CG2861 gene product from transcript CG2861-RA | 4,685302595 | - |
| CG13427 | CG13427 gene product from transcript CG13427-RA | 4,637894577 | - |
| CG5195 | CG5195 gene product from transcript CG5195-RA | 4,51569946 | - |
| CG8909 | CG8909 gene product from transcript CG8909-RC | 4,466839121 | + |
| CG7804 | CG7804 gene product from transcript CG7804-RA | 4,428759316 | - |
| NA | NA | 4,416718016 |  |
| Sp7 | Serine protease 7 | 4,390966643 | + |
| CG18765 | CG18765 gene product from transcript CG18765-RB | 4,32696177 | - |
| CG7458 | CG7458 gene product from transcript CG7458-RA | 4,292862163 | - |
| CG18631 | CG18631 gene product from transcript CG18631-RA | 4,16606905 | - |
| CG2893 | CG2893 gene product from transcript CG2893-RE | 4,146319718 | - |
| NA | NA | 4,115298235 |  |
| CG11915 | CG11915 gene product from transcript CG11915-RA | 4,098241244 | + |
| CG13397 | CG13397 gene product from transcript CG13397-RA | 4,067653884 | + |
| CG14529 | CG14529 gene product from transcript CG14529-RA | 4,063688498 | - |
| CG5783 | CG5783 gene product from transcript CG5783-RA | 4,060326161 | - |
| ine | inebriated | 4,056617739 | + |
| Ugt86Dj | CG15902 gene product from transcript CG15902-RA | 4,026795943 | + |
| RhoGAP100F | CG1976 gene product from transcript CG1976-RB | 4,00519857 | - |
| CG5707 | CG5707 gene product from transcript CG5707-RA | 3,968364336 | - |
| Cpr97Ea | Cuticular protein 97Ea | 3,960749935 | + |
| CG14958 | CG14958 gene product from transcript CG14958-RA | 3,931065823 | - |
| tipE | temperature-induced paralytic E | 3,927451177 | - |
| CG30217 | CG30217 gene product from transcript CG30217-RA | 3,912349383 | - |
| jdp | CG2239 gene product from transcript CG2239-RA | 3,903824046 | + |
| CG8008 | CG8008 gene product from transcript CG8008-RA | 3,899279097 | - |
| CG18304 | CG18304 gene product from transcript CG18304-RA | 3,881439115 | + |
| CG7582 | CG7582 gene product from transcript CG7582-RA | 3,862785621 | + |
| CG17928 | CG17928 gene product from transcript CG17928-RA | 3,856313875 | + |
| TyrR | Tyramine receptor | 3,794075733 | + |
| CG1625 | CG1625 gene product from transcript CG1625-RB | 3,789634492 | - |
| nrv3 | nervana 3 | 3,787488669 | - |
| CG14636 | CG14636 gene product from transcript CG14636-RA | 3,766828623 | + |
| CG15236 | CG15236 gene product from transcript CG15236-RB | 3,762457789 | + |
| CG8564 | CG8564 gene product from transcript CG8564-RA | 3,758274907 | - |
| Obp56a | Odorant-binding protein 56a | 3,751627044 | - |
| Hk | Hyperkinetic | 3,743068006 | - |
| CG17111 | CG17111 gene product from transcript CG17111-RA | 3,730699975 | - |
| GstE3 | Glutathione S transferase E3 | 3,719897342 | + |
| CG18536 | CG18536 gene product from transcript CG18536-RB | 3,697418976 | + |
| Ahcy89E | Adenosylhomocysteinase 89E | 3,681349453 | - |
| Cpr51A | Cuticular protein 51A | 3,628033034 | - |
| CG4329 | CG4329 gene product from transcript CG4329-RB | 3,567842727 | - |
| UGP | CG4347 gene product from transcript CG4347-RE | 3,557174299 | + |
| CG13640 | CG13640 gene product from transcript CG13640-RA | 3,532661976 | + |
| CG14866 | CG14866 gene product from transcript CG14866-RA | 3,523452478 | - |
| synaptogyrin | CG10808 gene product from transcript CG10808-RA | 3,50824775 | - |
| CG42260 | CG42260 gene product from transcript CG42260-RA | 3,507921627 | + |
| CG6300 | CG6300 gene product from transcript CG6300-RA | 3,495153951 | - |
| CG32703 | CG32703 gene product from transcript CG32703-RA | 3,4903677 | - |
| NA | NA | 3,48208538 |  |
| CG32132 | CG32132 gene product from transcript CG32132-RA | 3,457259147 | - |
| CG31551 | CG31551 gene product from transcript CG31551-RA | 3,402784152 | + |
| CG42329 | CG42329 gene product from transcript CG42329-RA | 3,375655466 | - |
| PGRP-LF | Peptidoglycan recognition protein LF | 3,374186559 | - |
| CG15143 | CG15143 gene product from transcript CG15143-RA | 3,36097792 | - |
| hiw | highwire | 3,356084357 | + |
| CG13065 | CG13065 gene product from transcript CG13065-RA | 3,349857226 | - |
| CG9083 | CG9083 gene product from transcript CG9083-RB | 3,318302842 | - |
| NA | NA | 3,311208223 |  |
| LvpH | Larval visceral protein H | 3,302853367 | - |
| CG6293 | CG6293 gene product from transcript CG6293-RA | 3,284157008 | - |
| Hsc70-1 | Heat shock protein cognate 1 | 3,239683547 | - |
| Mmp1 | Matrix metalloproteinase 1 | 3,137850843 | + |
| CG13733 | CG13733 gene product from transcript CG13733-RA | 3,123361529 | - |
| NA | NA | 3,108913118 |  |
| Ugt36Ba | CG13270 gene product from transcript CG13270-RA | 3,092406233 | - |
| CG3078 | CG3078 gene product from transcript CG3078-RA | 3,04808535 | + |
| CG11241 | CG11241 gene product from transcript CG11241-RB | 2,961973849 | + |
| NA | NA | 2,916893528 |  |
| NA | NA | 2,906997999 |  |
| CG8586 | CG8586 gene product from transcript CG8586-RA | 2,906524627 | - |
| CG32813 | CG32813 gene product from transcript CG32813-RD | 2,90079615 | + |
| nAchRbeta-64B | nicotinic Acetylcholine Receptor beta 64B | 2,888908355 | - |
| CG10559 | CG10559 gene product from transcript CG10559-RB | 2,887359178 | + |
| CG33012 | CG33012 gene product from transcript CG33012-RA | 2,839550299 | - |
| CG8147 | CG8147 gene product from transcript CG8147-RA | 2,822670445 | + |
| NA | NA | 2,818014523 |  |
| fzy | fizzy | 2,812675296 | + |
| CG32564 | CG32564 gene product from transcript CG32564-RA | 2,762163578 | - |
| CG33256 | CG33256 gene product from transcript CG33256-RA | 2,753048121 | - |
| Trl | Trithorax-like | 2,748885895 | + |
| psh | persephone | 2,744867915 | + |
| CG30456 | CG30456 gene product from transcript CG30456-RB | 2,714807572 | - |
| Cyp4ad1 | CG2110 gene product from transcript CG2110-RA | 2,700512949 | - |
| CG18446 | CG18446 gene product from transcript CG18446-RA | 2,687702078 | + |
| CG15056 | CG15056 gene product from transcript CG15056-RA | 2,637461685 | - |
| CG12972 | CG12972 gene product from transcript CG12972-RA | 2,624343185 | + |
| CG9232 | CG9232 gene product from transcript CG9232-RA | 2,583747105 | + |
| CG17264 | CG17264 gene product from transcript CG17264-RA | 2,528834222 | - |
| CG8086 | CG8086 gene product from transcript CG8086-RE | 2,522360193 | - |
| CG15021 | CG15021 gene product from transcript CG15021-RA | 2,501001797 | - |
| CG7381 | CG7381 gene product from transcript CG7381-RD | 2,474539478 | + |
| Ank2 | CG34416 gene product from transcript CG34416-RK | 2,459169195 | - |
| CG6114 | CG6114 gene product from transcript CG6114-RA | 2,451862558 | - |
| CG33120 | CG33120 gene product from transcript CG33120-RA | 2,451398735 | + |
| NA | NA | 2,422304878 |  |
| CG30497 | CG30497 gene product from transcript CG30497-RA | 2,413724746 | + |
| CG13046 | CG13046 gene product from transcript CG13046-RB | 2,410086086 | + |
| CG32694 | CG32694 gene product from transcript CG32694-RA | 2,376467074 | + |
| NA | NA | 2,341581548 |  |
| GluRIIA | Glutamate receptor IIA | 2,338312539 | - |
| NA | NA | 2,324058485 |  |
| GluRIIB | Glutamate receptor IIB | 2,311098596 | - |
| CG10126 | CG10126 gene product from transcript CG10126-RB | 2,305871688 | - |
| CG30275 | CG30275 gene product from transcript CG30275-RF | 2,280895649 | - |
| CG8303 | CG8303 gene product from transcript CG8303-RB | 2,269197984 | + |
| Faa | Fumarylacetoacetase | 2,212001327 | + |
| CG4325 | CG4325 gene product from transcript CG4325-RA | 2,185378348 | - |
| CG9460 | CG9460 gene product from transcript CG9460-RA | 2,155754926 | + |
| Cpr76Bd | Cuticular protein 76Bd | 2,154516986 | - |
| CG34400 | CG34400 gene product from transcript CG34400-RC | 2,144816537 | - |
| CG14572 | CG14572 gene product from transcript CG14572-RA | 2,133468409 | - |
| Ggamma1 | G protein gamma 1 | 2,022670126 | - |

**Downregulated genes**

| symbol | genename | fc | ChipSeq [26] |
| --- | --- | --- | --- |
| jhamt | juvenile hormone acid methyltransferase | -3,717427946 | - |
| Cpr78E | Cuticular protein 78E | -7,309433185 | - |
| CG32603 | CG32603 gene product from transcript CG32603-RA | -2,772898613 | - |
| cac | cacophony | -3,223708505 | - |
| SoxN | SoxNeuro | -2,400329639 | + |
| CG31248 | CG31248 gene product from transcript CG31248-RA | -4,37907393 | - |
| CG33469 | CG33469 gene product from transcript CG33469-RA | -2,586101751 | - |

Genes that change expression on GAGA overexpression ordered according to their fold change values. Only those with a fold change ≥ 2.0 are listed.
