# Supplementary material for: New insights for Drosophila GAGA factor in larvae
Source: R Soc Open Sci. 2015 Mar 18;2(3):150011. doi: 10.1098/rsos.150011 (PMC4448821; doi:10.1098/rsos.150011)
Supplement: Text for Suppl. Figures 1 to 7 [file rsos150011supp3.doc]

Supplementary material for Blanch et al.

Supplementary Figure 1.

Dorsal closure defects in embryos overexpressing GAGA with *pnr*GAL4. Four examples are shown. Arrows indicate the holes produced by dorsal closure defects that sometimes appeared more anterior (upper panels) and sometimes more posterior (lower panels). This phenotype had a full penetrance at 25°C.

Supplementary Figure 2.

Depletion had very little effect on expression of UBX. A. Immunostaining of imaginal disks from flies depleted of GAGA (*Nubb*GAL4; UAS-Dicer2> RNAiGAGA, upper panels) and control (*Nubb*GAL4; UAS-Dicer2, bottom panels) for GAGA (red), UBX (green) and DNA (by DAPI staining in blue). Wing (W), haltere (H), and leg (L) disks are indicated. All are confocal sections, and individual channels and merged channels are shown for clarity.

Supplementary Figure 3.

GAGA factor depletion causes DNA damage in wing disk cells. A. Confocal sections of wing disks depleted of GAGA factor in the wing pouch and immunostained for GAGA (in green), for γH2Av (in red) and for DNA (DAPI, in blue). Separate channels and a merge of them are shown for two independent samples. B. Confocal sections of wing disks depleted of GFP in the wing pouch and immunostained as above.

Supplementary Figure 4.

Salivary gland phenotype after limited GAGA factor overexpression. A. Control salivary glands (upper panels) and higher magnification of two nuclei from them stained with DAPI. B. Limited overexpression of GAGA factor for 13-15 h using *tub*GAL80^ts^; *Act*GAL4. Salivary glands (upper panels) and higher magnification of two nuclei from them stained with DAPI to visualize DNA.

Pictures were taken at the same magnification.

Supplementary Figure 5.

RT-PCR of total RNA extracted from disks overexpressing GAGA (induced) and control (non induced) after 13 h at 29°C using actGAL4. Upper panel indicates GAGA mRNA, central panel indicates Skl mRNA, and lower panel indicates U6 snRNA and was used as internal control. The rightmost two lanes show the signal of the assay for the same RNA samples without reverse transcription and indicates the absence of genomic DNA contamination.

Supplementary Figure 6.

GAGA factor activates *Skl* expression in S2 cells. A. Immunostaining of GAGA factor (in green) and SKL (in red) for a stably transfected S2 cell line for GAGA factor after induction (upper panels) and before (lower panels). DNA was stained with DAPI (in blue). B. Transient transfection of increasing amounts of GAGA factor results in a dose-dependent activation of a *Skl* promoter reporter construct in S2 cells. Results represent the mean of three independent experiments. Error bars indicate standard deviation.

Supplementary Figure 7.

More than apoptosis is required to account for GAGA overexpression phenotypes. A. *dpp*GAL4 mediated co-expression of GAGA and DIAP1 alleviates the phenotypes observed in wings (a, b) and legs (c to e). B. While undelayed GAGA overexpression mediated by *dpp*GAL4 led to lethality at pharates with loss of all legs [19], when GAGA overexpression started at 1^st^ instar larvae milder wing phenotypes and the absence of the leg phenotype appeared (not shown). C. While undelayed GAGA overexpression mediated by *ptc*GAL4 led to strong head defects that led to lethality at 2^nd^-3^rd^ instar larvae [19], GAGA overexpression started at 1^st^ instar larvae produced light wing phenotypes similar to the observed for *dpp*GAL4-mediated GAGA overexpression in B, and leg phenotypes similar to the observed in A (panels c to e). Arrows indicate abnormalities.
